# Metabolic plasticity, essentiality and therapeutic potential of ribose-5-phosphate synthesis in *Toxoplasma gondii*

**DOI:** 10.1038/s41467-024-47097-8

**Published:** 2024-04-08

**Authors:** Xuefang Guo, Nuo Ji, Qinghong Guo, Mengting Wang, Huiyu Du, Jiajia Pan, Lihua Xiao, Nishith Gupta, Yaoyu Feng, Ningbo Xia

**Affiliations:** 1 State Key Laboratory for Animal Disease Control and Prevention, South China Agricultural University, Guangzhou, China; 2 Guangdong Laboratory for Lingnan Modern Agriculture, Center for Emerging and Zoonotic Diseases, College of Veterinary Medicine, South China Agricultural University, Guangzhou, China; 3 Intracellular Parasite Education and Research Labs (iPEARL), Department of Biological Sciences, Birla Institute of Technology and Science, Pilani (BITS-P), Hyderabad, India; 4 Department of Molecular Parasitology, Faculty of Life Sciences, Humboldt University, Berlin, Germany

**Keywords:** Parasite biology, Parasite genetics

## Abstract

Ribose-5-phosphate (R5P) is a precursor for nucleic acid biogenesis; however, the importance and homeostasis of R5P in the intracellular parasite *Toxoplasma gondii* remain enigmatic. Here, we show that the cytoplasmic sedoheptulose-1,7-bisphosphatase (SBPase) is dispensable. Still, its co-deletion with transaldolase (TAL) impairs the double mutant’s growth and increases ^13^C-glucose-derived flux into pentose sugars via the transketolase (TKT) enzyme. Deletion of the latter protein affects the parasite’s fitness but is not lethal and is correlated with an increased carbon flux via the oxidative pentose phosphate pathway. Further, loss of TKT leads to a decline in ^13^C incorporation into glycolysis and the TCA cycle, resulting in a decrease in ATP levels and the inability of phosphoribosyl-pyrophosphate synthetase (PRPS) to convert R5P into 5′-phosphoribosyl-pyrophosphate and thereby contribute to the production of AMP and IMP. Likewise, PRPS is essential for the lytic cycle. Not least, we show that RuPE-mediated metabolic compensation is imperative for the survival of the *ΔsbpaseΔtal* strain. In conclusion, we demonstrate that multiple routes can flexibly supply R5P to enable parasite growth and identify catalysis by TKT and PRPS as critical enzymatic steps. Our work provides novel biological and therapeutic insights into the network design principles of intracellular parasitism in a clinically-relevant pathogen.

## Abstract (web summary)

Ribose-5-phosphate (R5P) is a precursor for nucleic acid biogenesis. Here, Guo and Ji et al. show that multiple routes can flexibly supply R5P to enable *Toxoplasma gondii* growth.

## Introduction

T*oxoplasma gondii* is an intracellular protozoan pathogen belonging to the phylum Apicomplexa. The parasite has a complex natural life cycle, shuttling between asexual and sexual stages^1^. Felines are the definitive (sexual) host for *T. gondii*, while other mammals and birds can serve as the intermediate (asexual) hosts. The parasite can reversibly switch between two infectious asexual stages, tachyzoites and bradyzoites, and can persist for the entire life of its host as tissue cysts containing bradyzoites^1,2^. Upon immune dysfunction, tissue cysts, primarily in the muscles and brain, may switch to tachyzoites, causing acute toxoplasmosis^3–5^. Since the current drugs, including pyrimethamine and sulfadiazine, are ineffective against tissue cysts, new antiparasitic targets and therapeutic options are needed. In this regard, metabolic pathways required for the reproduction and virulence of *T. gondii* have proven promising for developing antiparasitic drugs.

The pentose phosphate pathway (PPP) stemming from glycolysis is a fundamental route for producing glucose-derived carbon precursors for nucleotide synthesis and NADPH for anabolic reactions. We have recently reported that *T. gondii* has a functional PPP comprising several enzymes distributed in the cytoplasm and nucleus^6,7^. The PPP includes oxidative and nonoxidative branches, and the latter route consists of potentially reversible steps. Glucose enters into oxidative PPP via reactions catalyzed by glucose-6-phosphate dehydrogenase and 6-phosphogluconate dehydrogenase (6PGDH), generating ribulose-5-phosphate (Ru5P). Ru5P and other glycolytic metabolites are eventually converted into ribose-5-phosphate (R5P) via nonoxidative PPP. According to the current paradigm, R5P, an essential product of the PPP, is vital for nucleotide synthesis, cell growth and survival. Nonetheless, its subcellular homeostasis remains unclear during the asexual proliferation of *T. gondii*.

There are three putative pathways to generate R5P from glucose: the oxidative PPP, the nonoxidative PPP, and riboneogenesis^8^. The genetic and metabolic analysis of the latter two routes in *T. gondii* is still lacking. In mammalian cells^9^, the nonoxidative PPP relies on the transketolase (TKT) activity, catalyzing two reversible reactions: (a) inter-conversion of R5P and xylulose-5-phosphate (Xu5P) to/from sedoheptulose-7-phosphate (S7P) and glyceraldehyde-3-phosphate (GA3P), (b) catalysis of erythrose-4-phosphate (E4P) and Xu5P to/from fructose-6-phosphate (F6P) and GA3P. The relevance of TKT in *T. gondii* has not been examined. In addition, although transaldolase (TAL) is dispensable for the lytic cycle^6^, its contribution to the supply of R5P through nonoxidative PPP is unknown. Furthermore, although not regarded as a mainstream PPP enzyme, sedoheptulose-1,7-bisphosphatase (SBPase) can catalyze sedoheptulose-1,7-bisphosphate (SBP) to S7P, which is eventually converted to R5P by TKT^10^. Yet, the role of SBPase in parasite metabolism remains to be understood.

Here we show that irrespective of the routes of R5P biogenesis, it is used to generate 5′-phosphoribosyl-pyrophosphate (PRPP), a precursor for the nucleotides and amino acid biosynthesis^11,12^. The genome of *T. gondii* encodes a predicted phosphoribosyl-pyrophosphate synthetase (PRPS), potentially linking the PPP to the purine and pyrimidine biogenesis, but little is known about the physiological importance. In this work, we study the functional significance of *Tg*TKT, *Tg*TAL, *Tg*SBPase, *Tg*PRPS and *Tg*RuPE during the lytic cycle using a combinatorial approach involving gene mutagenesis, mutant phenotyping and metabolomics. Our data demonstrate exceptional plasticity of R5P biogenesis in tachyzoites while unraveling the parasite vulnerabilities for therapeutic intervention.

## Results

### SBPase expressed in the cytoplasm is dispensable for the tachyzoite growth

**Fig. 1:**
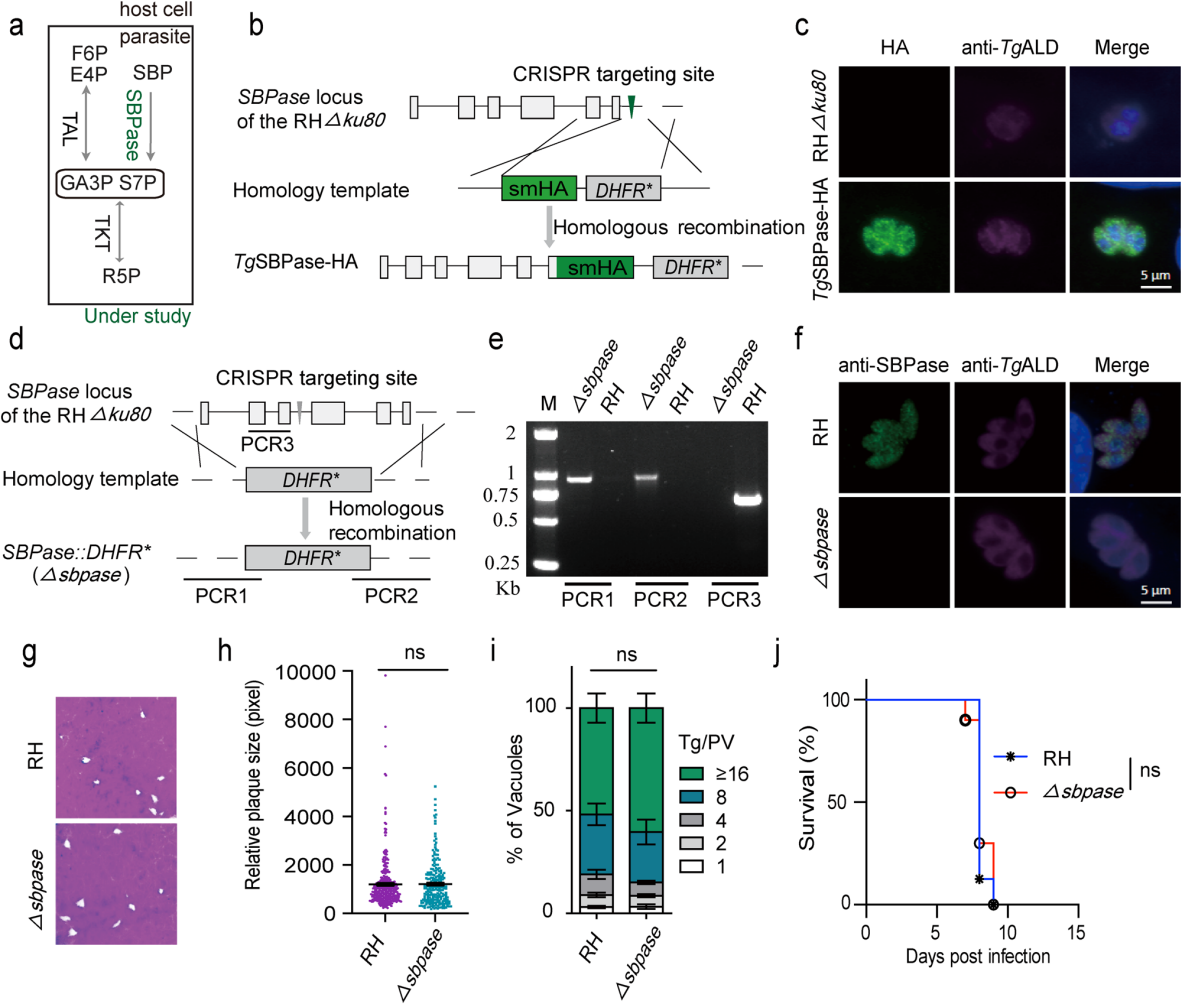
SBPase is dispensable for parasite growth. **a** Scheme of the R5P synthesis pathway in *T. gondii*, highlighting the SBPase enzyme. **b** Illustration depicting the 3′-genomic tagging of SBPase with a smHA epitope. **c** Co-localization of *Tg*SBPase-HA and *Tg*ALD in tachyzoites. The C-terminal smHA tagging of SBPase was achieved by CRISPR/Cas9-assisted site-specific integration in the RH*Δku80* strain. Scale bars = 5 μm. **d** Genetic deletion of *SBPase* by CRISPR/Cas9‐mediated genome editing. **e** PCR screening of a representative *Δsbpase* mutant. **f** Immunofluorescent staining confirming the loss of SBPase expression in the *Δsbpase* mutant. Scale bars = 5 μm. **g**, **h** Plaque assay to examine the overall fitness of the mutant. *n* = 3 experiments, means ± SEM; unpaired two-tailed Student’s *t*-test (ns, not significant, *p* = 0.9081). **i** The replication rates of the indicated strains (24 h infection, n = 3 assays, means ± SEM; ns, not significant, two-way ANOVA, *p* = 0.5561). **j** The virulence test in ICR mice. Animals were infected with 100 tachyzoites (10 mice/strain). Statistical significance was tested by log rank Mantel–Cox test (ns, not significant, *p* = 0.6701). Source data are provided as a Source data file.

We first investigated the subcellular expression of the HA-tagged SBPase, which was achieved by CRISPR/Cas9-assisted site-specific 3′-genomic integration in the RH*Δku80* strain (Fig. 1a, b). Immunostaining revealed that SBPase co-localized with the cytosolic marker protein, *Tg*ALD, in tachyzoites (Fig. 1c). To validate the cytoplasmic distribution of the endogenous SBPase, we generated polyclonal antisera against the recombinant protein purified from *E. coli* BL21 strain (Supplementary Fig. 1a). Yet again, immunofluorescence staining disclosed the presence of SBPase in the parasite cytosol (Supplementary Fig. 1d). Next, a *Δsbpase* mutant was engineered by CRISPR/Cas9‐mediated gene knockout strategy (Fig. 1d). The diagnostic PCRs screening using recombination-specific primers confirmed the deletion of *SBPase* in the parental RH*Δku80* strain (Fig. 1e). Loss of *SBPase* expression was confirmed by immunostaining (Fig. 1f). In plaque and replication assays, the *Δsbpase* mutant displayed normal growth, as judged by the plaque and vacuole size distribution when compared to the parental strain (Fig. 1g–i). Similarly, the ICR mice infected by fresh purified *Δsbpase* tachyzoites succumbed to death within 10 days of infection akin to the parental strain, indicating an unperturbed virulence of the mutant (Fig. 1j). These results show that SBPase is entirely dispensable for parasite growth in vitro and in vivo.

### The *ΔsbpaseΔtal* mutant displays impaired growth and adaptive regulation

**Fig. 2:**
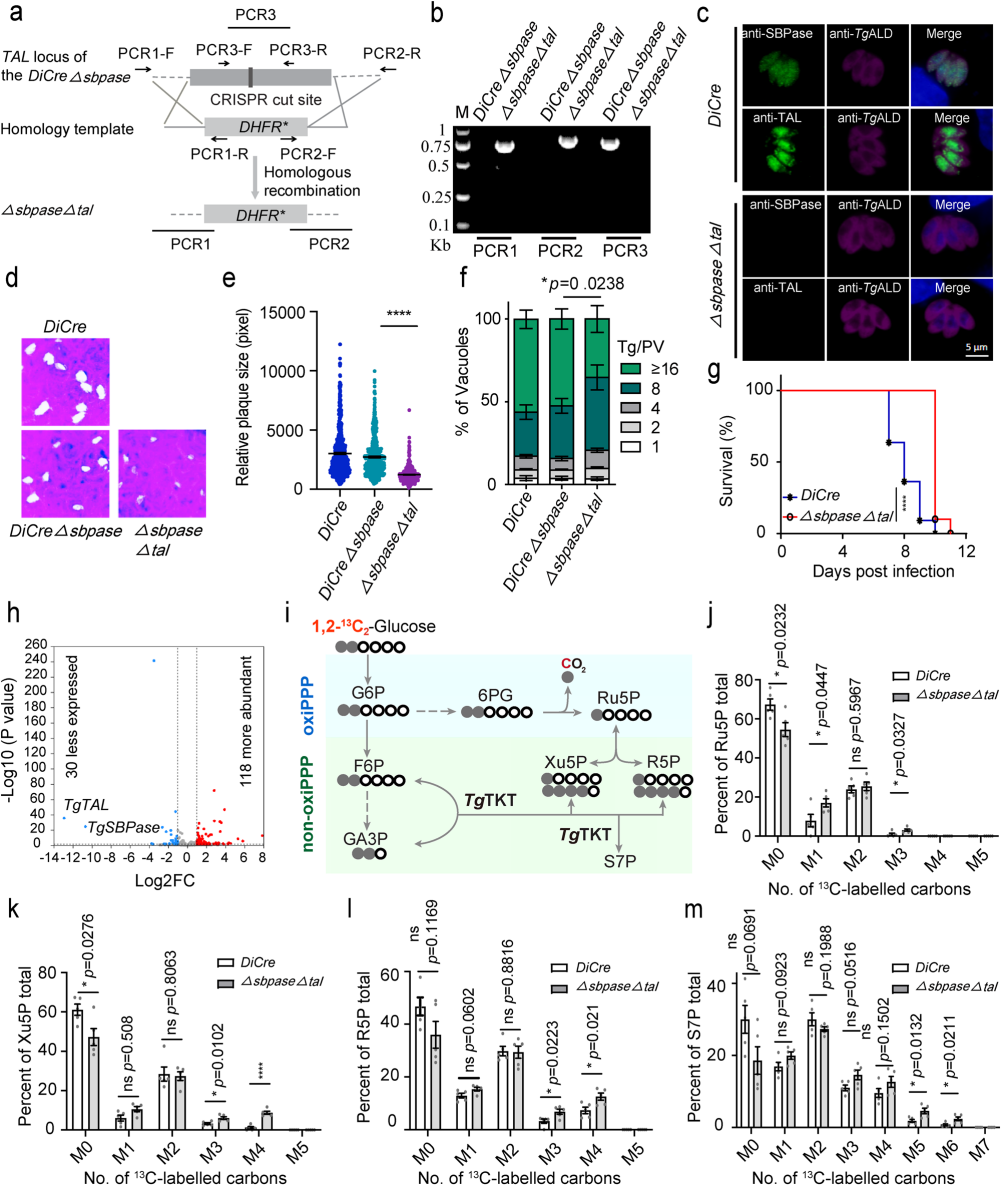
Phenotypic and carbon flux analyses of the *ΔsbpaseΔtal* mutant. **a** Schematics showing the construction of a *ΔsbpaseΔtal* mutant. **b** Diagnostic PCRs of a representative *ΔsbpaseΔtal* clone. **c** Immunostaining to validate the loss of SBPase and TAL expression in the *ΔsbpaseΔtal* mutant. Scale bars = 5 μm. **d**, **e** Plaque growth of the *ΔsbpaseΔtal* mutant in comparison to the *DiCreΔsbpase* or *DiCre* strains (*n* = 3 experiments, means ± SEM, unpaired two-tailed Student’s *t*-test; ****p < 0.0001. **f** Intracellular replication assay (24 h infection, *n* = 3 experiments, means ± SEM; two‐way ANOVA). **g** Survival curves of ICR mice infected with 100 tachyzoites of specified strains (*n* = 11 mice for *DiCre* and 10 mice for *ΔsbpaseΔtal* mutant). Statistical significance tested by log rank Mantel–Cox test. ****p < 0.0001. **h** Volcano plot showing differentially expressed genes in the *ΔsbpaseΔtal* mutant. Induced (red) and repressed genes (blue) with a log2 fold change ≥ 2 and *p* ≤ 0.05 are presented (*n* = 4 assays, Wale test *p* value in DESeq2, two-tailed). **i** Isotopomer formation and carbon flux schematics of 1,2-^13^C_2_-glucose. Gray balls correspond to ^13^C-labeled carbons, and white circles correspond to unlabeled ^12^C carbons. G6P: glucose-6-phosphate; F6P: fructose-6-phosphate; GA3P: glyceraldehyde-3-phosphate; 6PG: 6-phosphogluconate. **j**–**m** The *DiCre* and *ΔsbpaseΔtal* mutants were incubated in DMEM supplemented with 8 mM 1,2-^13^C_2_-glucose for 12 h. Metabolites were extracted, and the relative abundance of isotopologues was determined by LC-MS. M0 means an unlabeled C atom, while M1-M7 represents the number of carbon atoms in metabolites labeled with a ^13^C atom (*n* = 5 experiments, means ± SEM; ns, not significant; ****p < 0.0001; unpaired two-tailed Student’s *t*-test. Source data are provided as a Source data file.

No evident defect in the *Δsbpase* strain prompted us to speculate that F6P and E4P could be converted into S7P by transaldolase (TAL). We, therefore, constructed a *DiCreΔsbpase* strain (Supplementary Fig. 2a, b) and then deleted *TAL*, eventually generating a *ΔsbpaseΔtal* mutant (Fig. 2a). The screening PCRs (Fig. 2b), whole-genome sequencing (Supplementary Fig. 2c, d), semi-quantitative RT-PCR (Supplementary Fig. 2e) and immunofluorescence (Fig. 2c) assays confirmed the absence of *SBPase* and *TAL* in the double mutant. The *ΔsbpaseΔtal* strain formed significantly smaller plaques compared with the parental (*DiCre*) or progenitor (*DiCreΔsbpase*) strains, indicating a defective lytic cycle (Fig. 2d, e). Consistently, the double-deletion mutant exhibited impaired proliferation, as suggested by the enumeration of parasites replicating within parasitophorous vacuoles (Fig. 2f). We also observed a somewhat lower virulence of the mutant in comparison to the parental-infected animal group (Fig. 2g). In extended work, we complemented the *ΔsbpaseΔtal* mutant with an SBPase or TAL-expressing cassette, inserted at the *UPRT* locus by homologous recombination (Supplementary Fig. 3a). The complemented strains (comp*SBPase* and comp*TAL*) were obtained after 5-fluorodeoxyuridine (FUdR) selection and verified by diagnostic PCR and immunostaining (Supplementary Fig. 3b, c). Indeed, the phenotypic defect, as examined by the replication assay, was restored in the comp*SBPase* and comp*TAL* strain (Supplementary Fig. 3d). These results disclose a partial physiological redundancy of SBPase and TAL in tachyzoites.

To discern the adaptation in the *ΔsbpaseΔtal* strain, we analyzed its transcriptome compared to the parental (*DiCre*) parasites. In total, 30 transcripts were less expressed, while 118 were more abundant in the *ΔsbpaseΔtal* mutant, and as anticipated, the *SBPase* and *TAL* were among the top downregulated transcripts (Fig. 2h). Inactivation of the SBPase and TAL also led to reduced expression of BT1 family protein (BT1, TGGT1_201690) and 4-hydroxybenzoate polyprenyl transferase (TGGT1_259130), which are possibly involved in folate salvage and ubiquinone biosynthesis, respectively. Besides, the HECT-domain (ubiquitin-transferase)-containing protein (TGGT1_270580) was repressed in the mutant. These three genes have been assigned a negative phenotypic score in a genome-wide CRISPR screen (score = −1.28, −4.07, −2.98)^13^; their attenuated expression in the double mutant might therefore contribute to its growth impairment. The SBPase is a phosphatase that catalyzes the dephosphorylation of SBP to S7P and thereby supplies inorganic phosphate^8^. Loss of *SBPase* and *TAL* resulted in increased expression of haloacid dehalogenase family hydrolase domain-containing protein (HD, TGGT1_226100) and transporters (TGGT1_205265, TGGT1_268020, TGGT1_234570, TGGT1_235650), indicating increased phosphomonoester hydrolysis and nutrient acquisition as adaptive strategies of tachyzoites. The *ΔsbpaseΔtal* mutant also exhibited elevated levels of bradyzoite-specific SRS12D, suggesting a stress response (Supplementary Data 4). The qPCR verified the lower expression of BT1 and more abundant of HD and SRS12D transcripts in the *ΔsbpaseΔtal* mutants (Supplementary Fig. 2f). In extended work, we disrupted the *HD* and *SRS12D* in the *ΔsbpaseΔtal* mutant by CRISPR/Cas9-mediated insertion or deletion and generated the *ΔsbpaseΔtalΔhd* and *ΔsbpaseΔtalΔsrs12d* strains, respectively (Supplementary Fig. 4a–c). The triple knockout strains had no accentuated growth phenotype compared to the progenitor strain (Supplementary Fig. 4d–f). In addition, HD and SRS12D were also dispensable for parasite virulence in a mouse model (Supplementary Fig. 4g). The data indicated that the overexpression of HD and SRS12 is a type of adaptive regulation in the *ΔsbpaseΔtal* mutant that does not contribute to its survival.

**Fig. 3:**
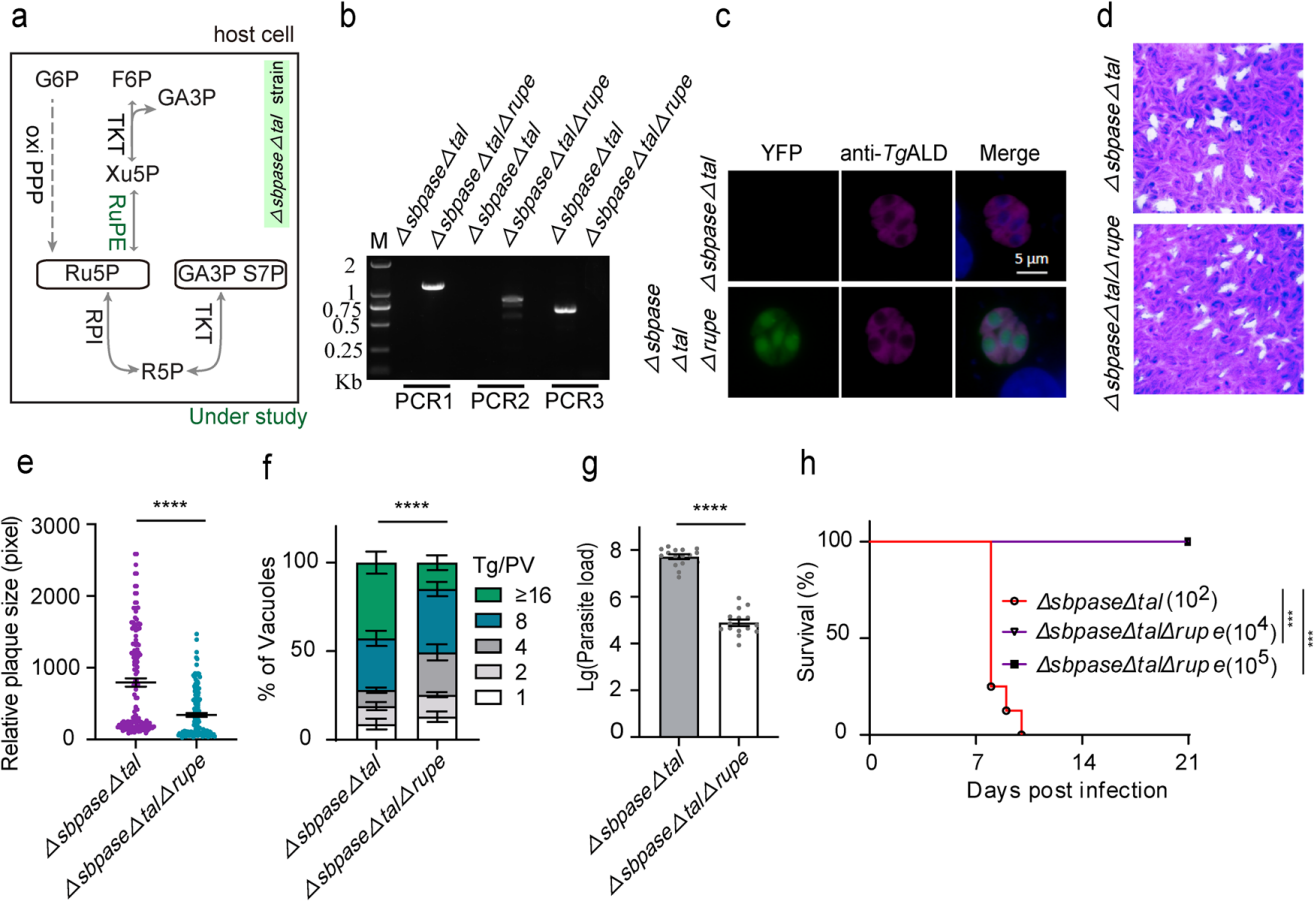
Deleting *RuPE* in the *ΔsbpaseΔtal* mutant harms parasite growth and virulence. **a** Scheme of the R5P synthesis pathway in the *ΔsbpaseΔtal* strain, highlighting the position of RuPE enzyme. **b** Diagnostic PCRs confirming the *ΔsbpaseΔtalΔrupe* mutant. **c** Immunostaining of the *ΔsbpaseΔtal* and *ΔsbpaseΔtalΔrupe* (YFP positive) strains with the rabbit anti-*Tg*ALD antibody. Scale bars = 5 μm. **d** Plaque assay assessing the comparative growth of the *ΔsbpaseΔtalΔrupe* mutant and its progenitor strain in HFF cells. **e** Plaque size based on the assay in **d** (*n* = 3 experiments, means ± SEM, unpaired two-tailed Student’s *t*-test; ****p < 0.0001). **f** Replication efficiency as determined by parasite distribution in the parasitophorous vacuoles. The number of parasites/vacuole was counted 24 h post-invasion (means ± SEM of three independent experiments, each with two replicates; ****p < 0.0001, two-way ANOVA). **g** Parasite burden in the peritoneal fluid of female ICR mice infected by the *ΔsbpaseΔtal* and *ΔsbpaseΔtalΔrupe* mutants (10^4^ tachyzoites per mouse). The parasite load was assessed by qPCR after 5 days of infection (5 mice/group; *n* = 3 assays; ****p < 0.0001, unpaired two-tailed Student’s *t*-test). **h** Virulence test in ICR mice infected with a dose of 10^2^ parasites of the *ΔsbpaseΔtal* strain (8 mice). The *ΔsbpaseΔtalΔrupe* mutant was also inoculated at higher doses (10^4^, 10^5^ parasites; 5 mice/group). Statistical significance was tested by log rank Mantel–Cox test. Compared with *ΔsbpaseΔtal* strain (***p = 0.0004). Source data are provided as a Source data file.

RuPE catalyzes the interconversion of R5P and Xu5P (Fig. 3a). Knockout of *RuPE* in the RH strain does not affect its growth and virulence^6^. We transfected the *RuPE*-specific CRISPR/Cas9 plasmid and the homology donor template containing YFP-DHFR* into the *ΔsbpaseΔtal* mutant to generate a triple knockout strain. After drug selection, fluorescent cell sorting, limiting dilution and diagnostic PCR, the *ΔsbpaseΔtalΔrupe* strain (YFP^+^) was obtained (Fig. 3b, c). The *ΔsbpaseΔtalΔrupe* mutant was evaluated by plaque, replication and parasite load assays (Fig. 3d–g). Indeed, deletion of *RuPE* in the *ΔsbpaseΔtal* strain significantly impaired the parasite growth. Moreover, while the *ΔsbpaseΔtal* strain caused animal death within 10 days, the *ΔsbpaseΔtalΔrupe* mutant was avirulent in the infected mice (Fig. 3h). These results suggest that the metabolic contribution of RuPE is critical for the in vitro and in vivo survival of the *ΔsbpaseΔtal* mutant.

### TKT is critical for tachyzoite survival and growth

To investigate the functional impact of the *SBPase* and *TAL* deletions on the pentose sugars synthesis, the intracellular tachyzoites of the *ΔsbpaseΔtal* and parental (*DiCre*) strains were cultured with 1,2-^13^C_2_-glucose for 12 h, followed by liquid chromatography-mass spectrometry (LC-MS) analysis (Supplementary Data 5). The distribution of isotope labeling in key PPP metabolites was analyzed (Fig. 2i). The M + 1 labeled Ru5P was increased in the *ΔsbpaseΔtal* mutant (Fig. 2j), suggesting a higher ^13^C flux into Ru5P through the oxidative PPP. The M + 4 labeled Xu5P and R5P and M + 5 and M + 6 S7P were also elevated in the *ΔsbpaseΔtal* strain (Fig. 2k–m). The tracer labeling reflected that TKT catalyzes F6P and G3P to produce Xu5P via the nonoxidative PPP, thereby increasing the flux of glucose-derived carbon into pentose sugars of the double mutant. As such, TKT facilitating the reversible transfer of a keto unit between donors and acceptor substrates appeared to be a central enzyme in the nonoxidative branch of the PPP.

**Fig. 4:**
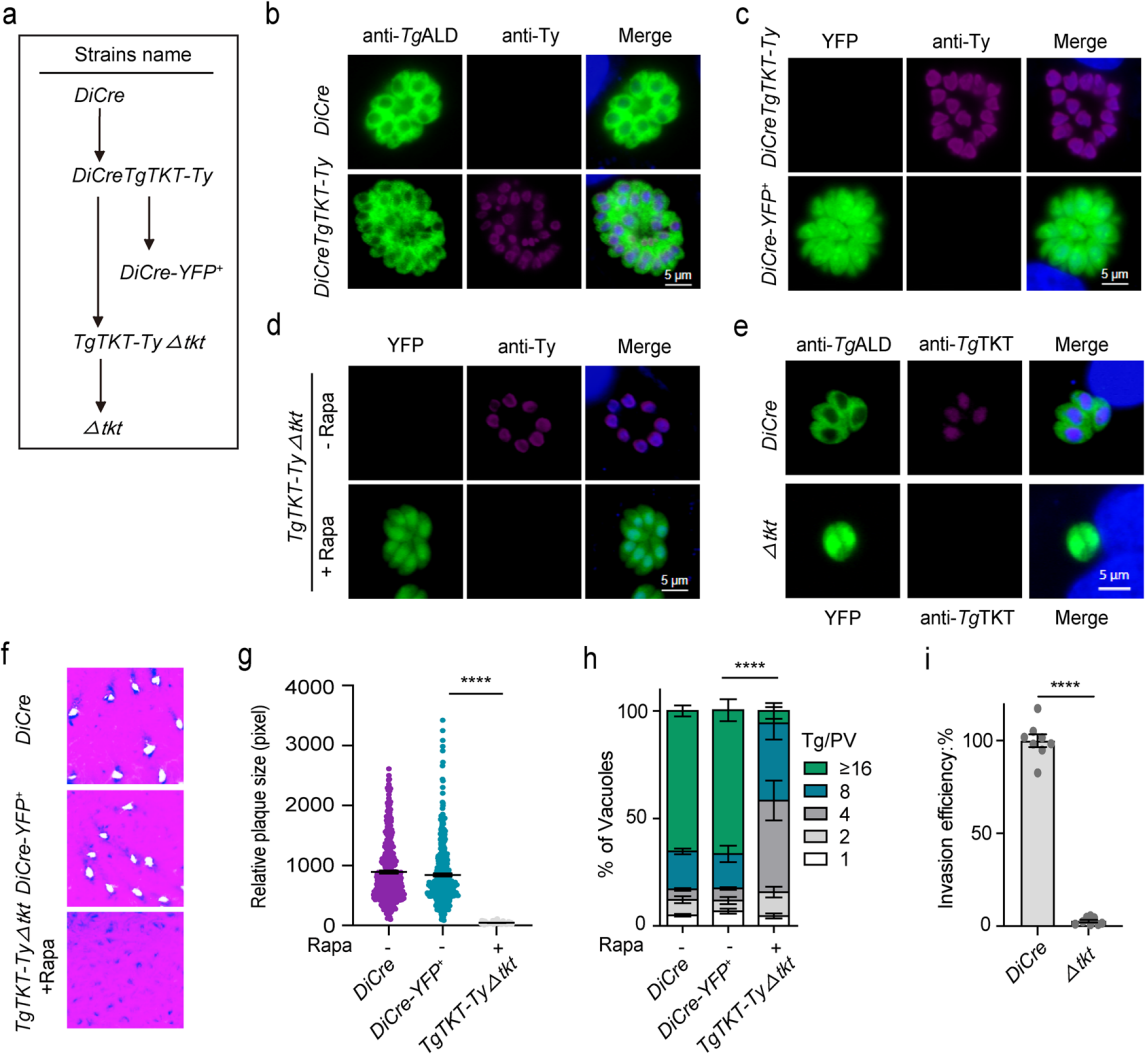
TKT is essential for the lytic cycle of parasites. **a** Engineering of TKT strains. **b** Immunostaining of TKT in the *DiCreTgTKT*-*Ty* strain. Parasites were stained with anti-Ty and anti-*Tg*ALD antibodies. Scale bars = 5 μm. **c** Immunofluorescence staining of *DiCreTgTKT-Ty* and *DiCre-YFP*^*+*^ with anti-Ty antibody. Scale bars = 5 μm. **d** Expression of *TgTKT-Ty* in *TgTKT-TyΔtkt* strain. *TgTKT-TyΔtkt* parasites were cultured without or with rapamycin for 4 days and then stained with anti-Ty antibody. Scale bars = 5 μm. **e** Expression of TKT in the *Δtkt* mutant. The parental *DiCre* strain was stained with mouse anti-*Tg*TKT and rabbit anti-*Tg*ALD, whereas the *Δtkt* strain (YFP positive) was stained with mouse anti-*Tg*TKT. Scale bars = 5 μm. **f-h** Plaque and replication assays with indicated strains. The *TgTKT-TyΔtkt* tachyzoites were treated with rapamycin for 4 d to deplete *TKT* before analysis. *n* = 3 experiments, means ± SEM; unpaired two-tailed Student’s *t*-test (****, *p* < 0.0001). Intracellular replication assay (24 h infection; *n* = 3 assays, means ± SEM; ****, *p* < 0.0001, two‐way ANOVA). **i** Invasion efficiency of the *DiCre* and *Δtkt* strains, as determined by two-color staining to distinguish invaded *vs* non-invaded parasites (*n* = 4 experiments, each with two technical replicates, means ± SEM; ****p < 0.0001, unpaired two-tailed Student’s *t*-test). Source data are provided as a Source data file.

We examined the expression, localization and importance of TKT in tachyzoites. The open reading frame of TKT was amplified by PCR, cloned into the *pCold* vector, and expressed in *E. coli* (BL21). The protein expression was analyzed by SDS-PAGE (Supplementary Fig. 1c), and polyclonal antibodies against the purified recombinant TKT were produced in the ICR mice. Subsequent immunofluorescence assay revealed that the native TKT localizes in the parasite nucleus (Supplementary Fig. 1f), which resonates with the subcellular distribution of the C-terminally-tagged TKT^6^. To determine the functional relevance of TKT, we first attempted to delete the gene by CRISPR/Cas9-assisted double homologous recombination. However, no knockout mutants could be obtained after transgenic selection, implying that TKT might be vital for parasite growth. We, therefore, constructed a *DiCreTgTKT-Ty* mutant, in which the *UPRT* locus was replaced by the 3′Ty-tagged ORF of TKT (Supplementary Fig. 6a, Fig. 4a). The integration at the desired locus and expression of TKT in the parasite nucleus were verified by crossover-specific PCR and immunofluorescence methods (Supplementary Fig. 6b, Fig. 4b). The clonal mutants (*DiCre-YFP*^*+*^) were generated by rapamycin-mediated *TKT* deletion in *DiCreTgTKT-Ty* strain and analyzed by diagnostic PCR and immunofluorescence assays (Supplementary Fig. 6c, Fig. 4c).

In the next step, we engineered a *TgTKT-TyΔtkt* strain by deploying a pyrimethamine-resistant dihydrofolate reductase thymidylate synthase (*DHFR*-TS^*^) selection marker, followed by rapamycin treatment (Supplementary Figs. 6a, d and Fig. 4d). Subsequently, the *TgTKT-TyΔtkt* mutant pretreated with rapamycin for 4 days was tested for its ability to form plaques in host cell monolayers. The *DiCre* and *DiCre-YFP*^*+*^ strains, phenocopying each other, served as suitable controls. The rapamycin-treated *TgTKT-TyΔtkt* mutant, however, did not form any plaques even 7 days after infection (Fig. 4f–g). Similarly, its replication was impaired compared to the controls (Fig. 4h).

### The *Δtkt* mutant is avirulent in mice and confers immunity to challenge infection in mice

**Fig. 5:**
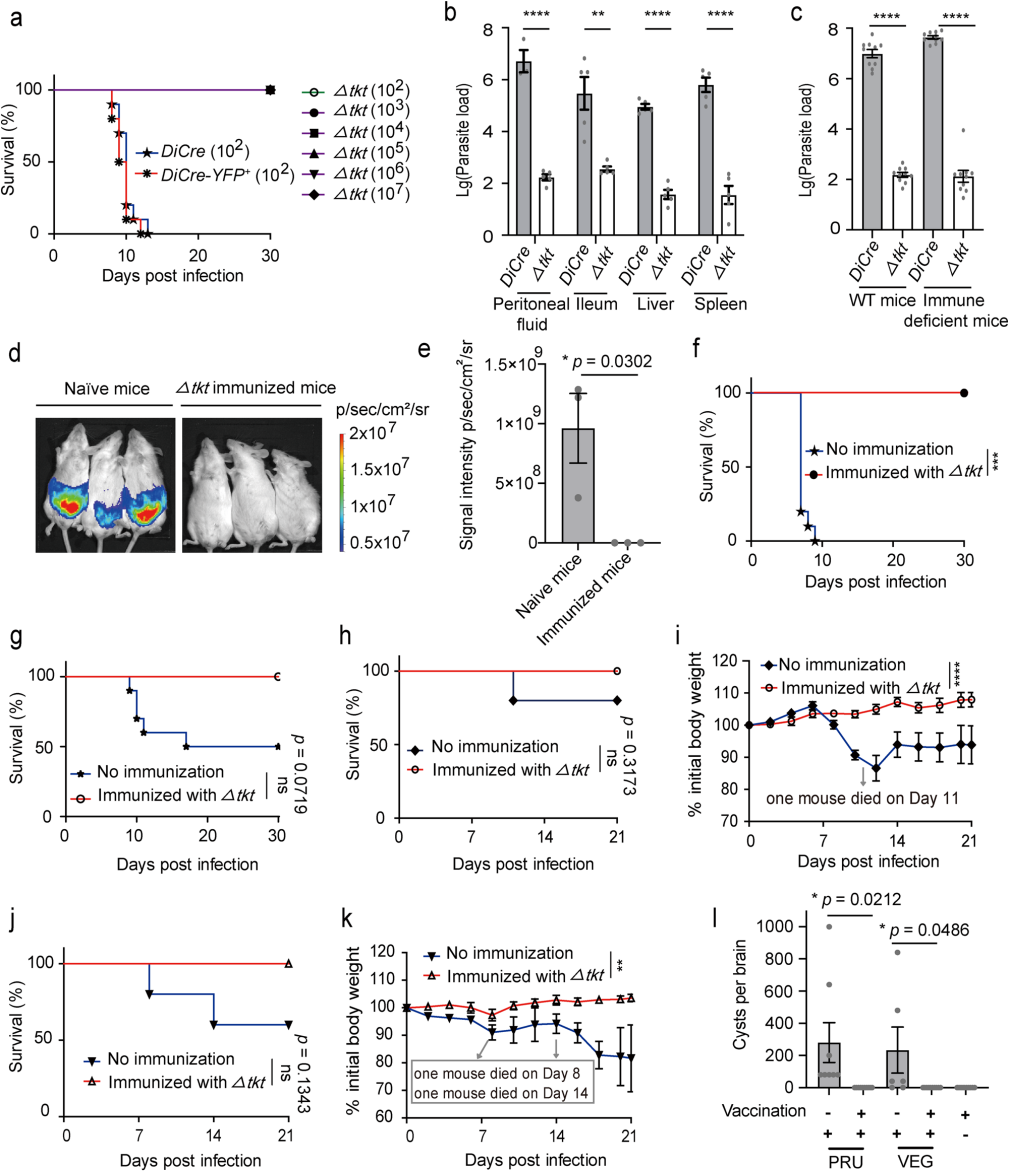
In vivo relevance of TKT and vaccination potential of the *Δtkt* mutant. **a** Virulence test in ICR mice infected with a dose of 10^2^ parasites of the specified strains (10 mice). The *Δtkt* mutant was also inoculated at higher doses (10^3^, 10^4^, 10^5^, 10^6^, 10^7^ parasites; 5–8 mice/group). **b** Parasite burden in the peritoneal fluid, ileum, liver and spleen of female ICR mice infected with 10^4^ parasites of the *DiCre* or *Δtkt* strains. The parasite load was determined 5 days post-infection by qPCR (*n* = 5 mice/group; means ± SEM; **p = 0.0018, ****p < 0.0001, unpaired two-tailed Student’s *t*-test). **c** Propagation of *DiCre* and *Δtkt* strains in ICR and Balb/c-nu mice infected with 10^4^ parasites. The peritoneal fluid was analyzed by qPCR 5 days post-infection (*n* = 5 animals/group; two independent experiments, means ± SEM; ****p < 0.0001, unpaired two-tailed Student’s *t*-test). **d**, **e** Infection of the *Δtkt*-vaccinated and naïve mice with RH-*Luc* strain (10^4^). The parasite load in the abdominal cavity was assessed by bioluminescence imaging (*n* = 3 independent experiments; means ± SEM, Student’s *t*-test). **f**–**k** Immunization of mice with the *Δtkt* mutant and challenge infection. Seven-week-old ICR mice were immunized with 10^2^, 10^3^ or 10^4^ parasites, and 21 or 30 days later, they were intraperitoneally injected with 10^4^ parasites of the *DiCre* (**f**, *n* = 5/10 mice/strain; statistical significance tested by log rank Mantel–Cox test; ***p = 0.0001), ME49 (**g**, *n* = 5/10 mice/strain; ns, not significant), PRU (**h**, *n* = 5 mice/strain; ns not significant) (**i**, *n* = 5 experiments, means ± SEM; ****p < 0.0001, two‐way ANOVA) or VEG (**j**, *n* = 5 mice/strain; ns not significant) (**k**, *n* = 5 experiments, means ± SEM; **p = 0.0027, two‐way ANOVA) strains. The survival was monitored for an additional 21 or 30 days. Unimmunized (naïve) mice were included as the control group. **l** Vaccination with *Δtkt* prevented the formation of cysts in the mice. The *Δtkt*-immunized and naïve mice were infected with PRU or VEG strains and then sacrificed 21 days after the challenge, and the number of *Toxoplasma* cysts in the brain was counted by DBA staining with two technical replicates (*n* = 5 mice for *Δtkt-*immunized group, *n* = 4 mice for naïve group infected with PRU. *n* = 3 mice for naïve group infected with VEG; means ± SEM, Student’s *t*-test). Source data are provided as a Source data file.

To evaluate the in vivo relevance of TKT, a clonal mutant (*Δtkt*) was produced from rapamycin-treated *TgTKT-TyΔtkt* strain and validated by PCR, RT-PCR and immunostaining assays (Supplementary Fig. 6e, f and Fig. 4e). Surprisingly, the *Δtkt* mutant was viable in prolonged culture (e.g., plaque assay for two-weeks) (Supplementary Fig. 6g) despite severe defects in the parasite replication and host-cell invasion (Fig. 4h–i). We compared its virulence to the parental *DiCre* and *DiCre-YFP*^*+*^ strains by intraperitoneal injection of the ICR mice (100 parasites/animal). As expected, the control strains inflicted a lethal phenotype in nearly all animals within 12 days. In contrast, the *Δtkt*-infected mice did not show any clinical signs and survived even at much higher doses (up to 10^7^ parasites/mouse) (Fig. 5a), indicating a remarkable attenuation of virulence. The *DiCre* and *Δtkt* strains (10^4^ tachyzoites/mouse) were also examined for in vivo proliferation in the peritoneal fluid, ileum, liver, and spleen by quantitative PCR five days post-infection. Indeed, the parasite burdens in mice infected with *Δtkt* mutant were lower than those infected with the *DiCre* strain (Fig. 5b), revealing a vital role of TKT in the in vivo development of tachyzoites.

Our additional work tested whether the host immune response underlies the observed attenuated growth of the *Δtkt* mutant in mice. We infected immune‐deficient Balb/c-nu mice (10^4^ parasites/animal) and quantified the parasite burden in the peritoneal fluid. Similar to our data in the immune-competent ICR mice, the *Δtkt* tachyzoites were almost undetectable in the Balb/c-nu mice (Fig. 5c). These data suggest the intrinsic inability of the *Δtkt* mutant to propagate and establish in vivo infection. Considering that the *Δtkt* could be cultured in vitro but failed to proliferate in mice, we subsequently explored the potential of the genetically attenuated *Δtkt* strain as a whole-cell vaccine using the mouse model. As anticipated, the *Δtkt* immunization enabled rapid clearance of the challenge infection by the RH*-Luc* tachyzoites (Fig. 5d, e). Moreover, all *Δtkt*-vaccinated mice survived following challenge infection by tachyzoites of type I (*DiCre*), type II (ME49 and PRU) or type III (VEG) strains. In contrast, non-immunized naïve mice exhibited obvious clinical signs and weight loss (Fig. 5f–k). Examination of the cyst burden in the *Δtkt*-immunized and naïve mice by DBA staining revealed about 250 cysts/brain in the non-immunized mice infected with the PRU or VEG strains, whereas none in the *Δtkt*-immunized mice (Fig. 5l). These results underscore the therapeutic value of TKT and *Δtkt* mutant as a drug target and protective immunity, respectively.

### Deletion of *TKT* results in increased carbon flux through oxidative PPP

**Fig. 6:**
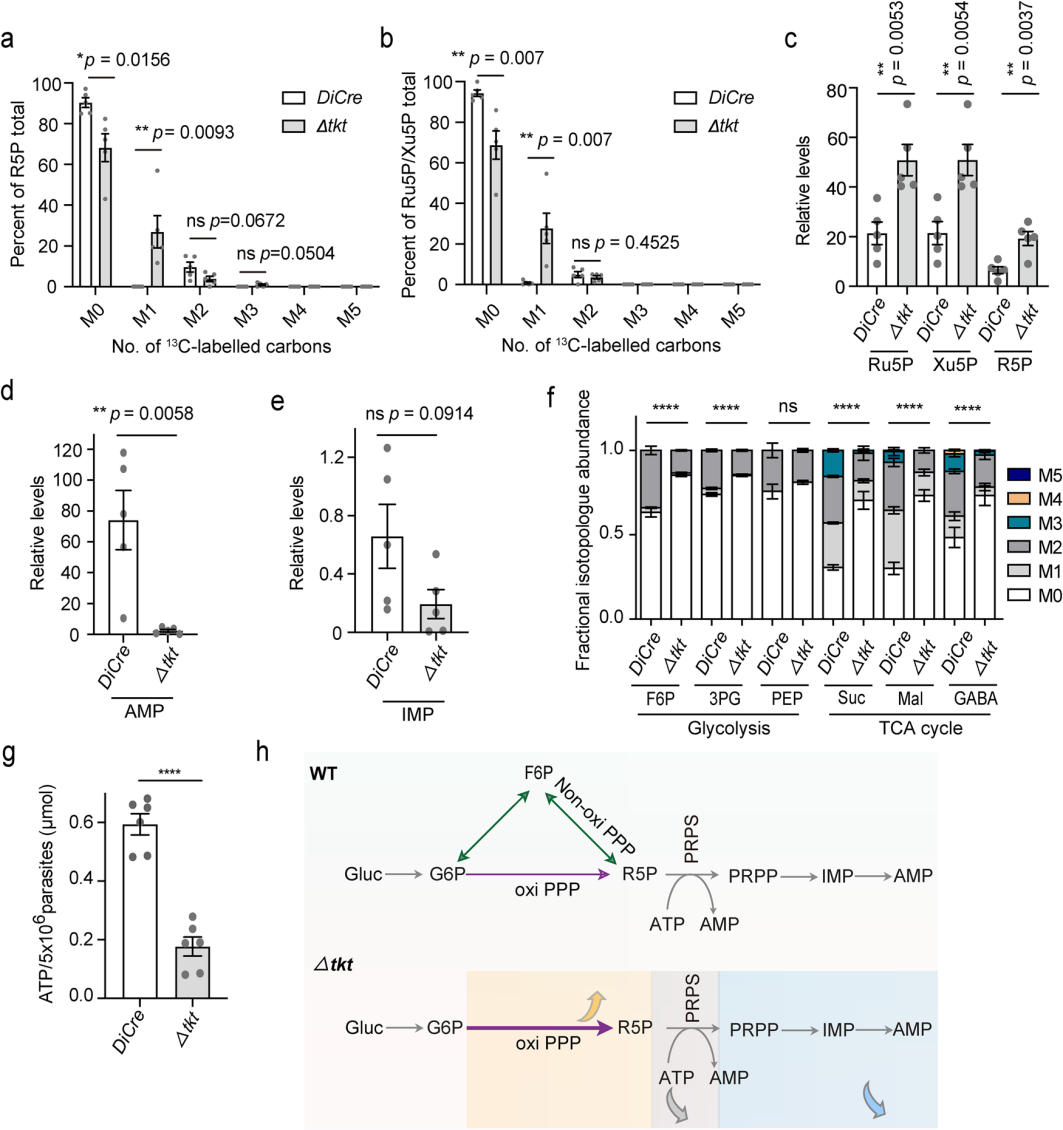
The *Δtkt* mutant displays a higher carbon flux through oxidative PPP. **a**, **b** Extracellular parasites of the *DiCre* and *Δtkt* strains were incubated for 4 h in a medium with 8 mM 1,2-^13^C_2_-glucose. The incorporation of ^13^C into pentose sugars was measured by UHPLC-HRMS (*n* = 5 experiments, means ± SEM; unpaired two-tailed Student’s *t*-test). **c**–**e** Intracellular *DiCre* and *Δtkt* parasites grown under standard tissue culture conditions. UHPLC-HRMS was used to analyze Ru5P, Xu5P, R5P, AMP and IMP (*n* = 5, means ± SEM; unpaired two-tailed Student’s *t*-test). **f** Intracellular *DiCre* and *Δtkt* parasites were incubated for 12 h in a medium with 8 mM 1,2-^13^C_2_-glucose. The incorporation of ^13^C into glycolysis and TCA metabolites was measured by UHPLC-HRMS. F6P fructose-6-phosphate, 3PG 3-phosphoglycerate, PEP phosphoenolpyruvate, Suc succinate, Mal malate, GABA gamma-aminobutyric acid. M0-M5 represents the number of carbon atoms in selected ^13^C-labeled metabolites. (*n* = 5, means ± SEM; ns not significant; *p* = 0.14, ****p < 0.0001, two-way ANOVA). **g** The ATP levels in the *DiCre* and *Δtkt* parasites were measured by the ATP assay kit. Means ± SEM (*n* = 3 assays, each with two technical replicate; ****p < 0.0001, unpaired two-tailed Student’s *t*-test). **h** Wild type (WT) strain can produce R5P via oxiPPP and non-oxiPPP, whereas the *Δtkt* mutant relies solely on oxiPPP for its R5P supply. *Δtkt* mutants have reduced levels of ATP, resulting in the inability of R5P to be catalyzed by PRPS to PRPP, leading to reduced levels of AMP and IMP and the accumulation of R5P. Gluc glucose, G6P glucose-6-phosphate. Source data are provided as a Source data file.

To explain the *Δtkt* viability in culture, we measured the 1,2-^13^C_2_ glucose-derived carbon flux through PPP in extracellular parasites using LC-MS (Supplementary Data 6). The level of M + 1 labeled R5P was higher in the *Δtkt* mutant compared to the parental strain (Fig. 6a). Likewise, Xu5P and Ru5P containing one ^13^C were also increased in the mutant (Fig. 6b). By contrast, we found that the incorporation of ^13^C into glycolysis and TCA cycle intermediates was reduced in the *Δtkt* strain (Supplementary Fig. 10). These data imply a switch to oxidative PPP as the primary route for pentose sugar synthesis following the loss of *TKT* in parasites. The above results are consistent with two effective routes of R5P production from glucose, i.e., oxidative and nonoxidative branches of PPP.

The key enzyme of the oxidative PPP in *T. gondii* is 6PGDH2, whereas the nonoxidative route relies on the RPI and TKT activity (ref. ^6^, this work). To compare the effect of individual deletions on parasite growth, we performed the plaque and qPCR assays using the *Δ6pgdh2*, *Δrpi*, *Δtkt* and *DiCre* strains under standard culture conditions. Plaques formed by the *Δ6pgdh2* mutant were significantly smaller than the control *DiCre* strain but more prominent than the *Δrpi* tachyzoites, whereas knockout of *TKT* did not result in visible growth (Supplementary Fig. 7a, b). The growth rates of the indicated strains were confirmed by qPCR (Supplementary Fig. 7c). Likewise, the *Δtkt* mutant exhibited much smaller parasitophorous vacuoles harboring only 1 or 2 parasites due to prolonged replication (Supplementary Fig. 7d). Collectively, our data suggest that both oxidative and nonoxidative PPP routes are required to support parasite growth. Still, none appears to be essential, and R5P can be produced flexibly via redundant metabolic pathways.

### The *Δtkt* mutant undergoes stage switching with an adaptive regulation of its proteome

Having determined the impact of *TKT* deletion on the lytic cycle and given that R5P is needed for the nucleotide and amino acid syntheses, we performed an MS-based proteomic analysis to gain added insight into parasite metabolism. The quantitative proteomics enabled the identification and relative quantification of 3296 *Toxoplasma* proteins. In total, 350 proteins were downregulated, while 326 proteins were upregulated in the *Δtkt* strain (Supplementary Fig. 8a). Subsequently, we analyzed the putative localization of proteins and found that many nuclear proteins were impacted upon the deletion of *TKT* (Supplementary Fig. 8b). The KEGG pathway enrichment revealed a perturbation of proteins mainly involved in purine metabolism, RNA polymerase and ribosome biogenesis (Supplementary Fig. 8c). We noted a striking decrease in specific RPB proteins involved in transcription and translation initiation. A few Rab and PIP5K proteins associated with autophagy and endocytosis were increased (Supplementary Fig. 8d), implying adaptive modulation in the *Δtkt* strain. Besides, the expression of some micronemal and rhoptry proteins declined in the mutant, coherent with its invasion defect (Supplementary Fig. 8e). Not least, akin to the *Δrpi* strain^6^, we found tachyzoite-specific SRS repressed, while selected bradyzoite-specific SRS were increased upon *TKT* deletion. The qPCR of selected genes confirmed induction of *SRS44* and *Rab2* and repression of *ROP39, MIC4* and *MIC6* transcripts in the *Δtkt* strains (Supplementary Fig. 8f). Collectively, these results (Supplementary Data 10) suggest that the parasite undergoes an adaptive regulation of its proteome upon impairment of ribose-5-phosphate synthesis.

To ascertain the adaptive changes upon nucleotide limitation caused by *TKT* deletion, periodic acid-Schiff (PAS) staining of polysaccharides was performed. We observed an accumulation of amylopectin in the *Δtkt* mutants. In contrast, it was undetectable in the *DiCre* strain (Supplementary Fig. 9a). We then tested the presence of cyst formation by staining with rhodamine-labeled Dolichos Biflorus Agglutinin (DBA) and observed a higher proportion of DBA-positive vacuoles in the mutant (Supplementary Fig. 9b, c). Besides, the length and width of the *Δtkt* mutant, scored after *Tg*GAP45 immunostaining, was significantly reduced compared to the parental strain (Supplementary Fig. 9d–f). In summary, *TKT* depletion causes adaptive reprogramming and bradyzoite formation, possibly due to nucleotide limitation.

### Utilization of ribose-5-phosphate is essential for tachyzoite survival

We next examined the abundance of R5P in the *ΔsbpaseΔtal* and *Δtkt* mutants. The double-deletion of *SBPase* and *TAL* or knockout of *TKT* led to a significant accumulation of R5P (Fig. 6c, Supplementary Data 7; Supplementary Fig. 5a, Supplementary Data 5). Further, Xu5P and Ru5P were also more abundant in the *Δtkt* mutant compared to its parental strain (Fig. 6c, Supplementary Data 7). However, adenosine monophosphate (AMP) and inosine monophosphate (IMP) were reduced after the deletion of *TKT* (Fig. 6d, e, Supplementary Data 7) and in the double mutant (Supplementary Fig. 5b, c, Supplementary Data 9). Likewise, the *ΔsbpaseΔtal* and *Δtkt* mutants harbored significantly lower amounts of ATP than the parental strain (Fig. 6g, Supplementary Fig. 5d). Not least, similar to the metabolic flux analysis using extracellular parasites (Supplementary Fig. 10), ^13^C incorporation into glycolysis and TCA cycle intermediates of the intracellular parasites of the *Δtkt* mutant was decreased (Fig. 6f, Supplementary Data 8). These results suggest that excess of R5P may not be efficiently utilized by PRPS, possibly due to ATP deprivation, in *ΔsbpaseΔtal* and *Δtkt* mutant (Fig. 6h).

**Fig. 7:**
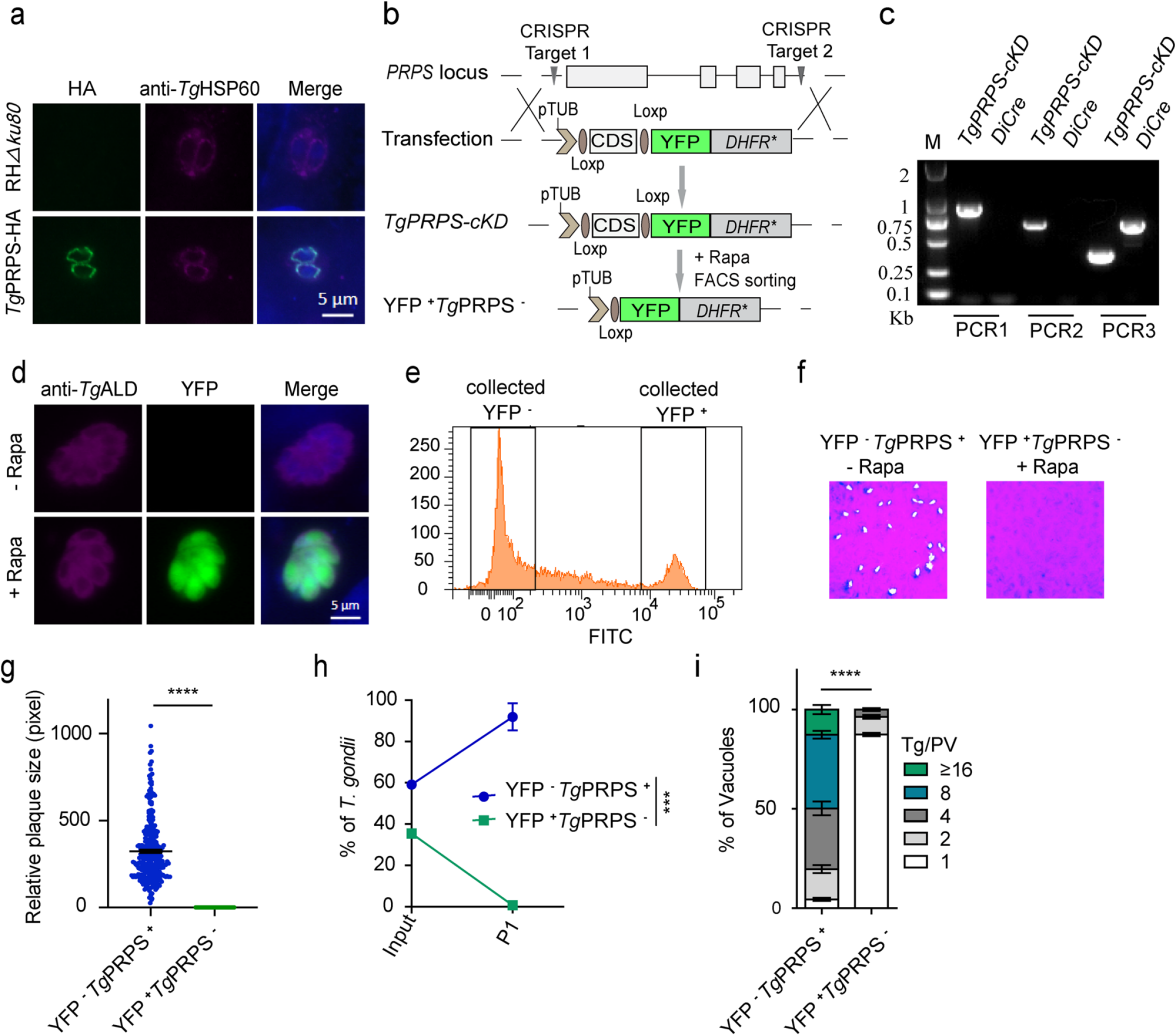
Knockout of *PRPS* impairs parasite proliferation. **a** Immunolocalization of *Tg*PRPS-HA with HSP60 (a mitochondrial marker, 1:1000). The transgenic strain was generated by CRISPR-assisted 3′-genomic tagging. Scale bars = 5 μm. **b** Replacement of *PRPS* in the *DiCre* strain by CRISPR/Cas9-mediated homologous recombination. **c** Diagnostic PCRs on a conditional mutant (*TgPRPS-cKD*). **d** Immunofluorescence staining of *Tg*ALD and expression of YFP in the *TgPRPS-cKD* mutant incubated with rapamycin for 5 days. Scale bars = 5 μm. **e** Flow cytometry of YFP expression in rapamycin-treated mutant. **f**, **g** Plaque assay of the *TgPRPS-cKD* strain (−/+ rapamycin, 5 days). (*n* = 3 experiments, means ± SEM, ****p < 0.0001, unpaired two-tailed Student’s *t*-test). **h** Competition assay (−/+ rapamycin, 5 days) (*n* = 3 experiments; means ± SEM; P1, ***p = 0.0002, unpaired two-tailed Student’s *t*-test). **i** Replication rates of the *TgPRPS-cKD* mutant (−/+ rapamycin, 5 days). Parasites expressing YFP (YFP^+^*Tg*PRPS^−^) were sorted by FACS. Intracellular tachyzoites (24 h infection) replicating in parasitophorous vacuoles were counted (Tg/PV; *n* = 3 assays, means ± SEM; ****p < 0.0001, two-way ANOVA). Source data are provided as a Source data file.

Our final experiments focused on PRPS to fathom the physiological importance of R5P in tachyzoites. PRPS catalyzes the reaction of R5P with ATP to produce PRPP and AMP. We first fused the C-terminus of PRPS with a spaghetti monster hemagglutinin (smHA) epitope by 3′-insertional tagging of the gene, followed by immunofluorescence staining. PRPS was expressed in the tachyzoite mitochondrion, co-localizing with the organelle marker HSP60 (Fig. 7a). We then generated a rapamycin-inducible mutant of PRPS using the DiCre system because its deletion by gene replacement could not be achieved (Fig. 7b). A conditional knockdown mutant of PRPS (*TgPRPS*-*cKD*, YFP^-^*Tg*PRPS^+^) was obtained after pyrimethamine selection and confirmed by diagnostic PCR and immunostaining (Fig. 7c, d). We isolated YFP^+^*Tg*PRPS^-^ parasites after five days of rapamycin treatment by fluorescent cell sorting (Fig. 7e). The plaque, growth competition and replication assays using the YFP^-^*Tg*PRPS^+^ and YFP^+^*Tg*PRPS^-^ tachyzoites were performed. Knockout of PRPS after rapamycin treatment led to substantial defects, as seen by the near-absence of plaques and the loss of mutants within one passage (Fig. 7f–h). The *PRPS*-null tachyzoites could almost not divide (Fig. 7i) and did not survive over two weeks after culturing in normal conditions. These results demonstrate that downstream utilization of ribose-5-phosphate by PRPS is critical for tachyzoite growth and survival.

## Discussion

**Fig. 8:**
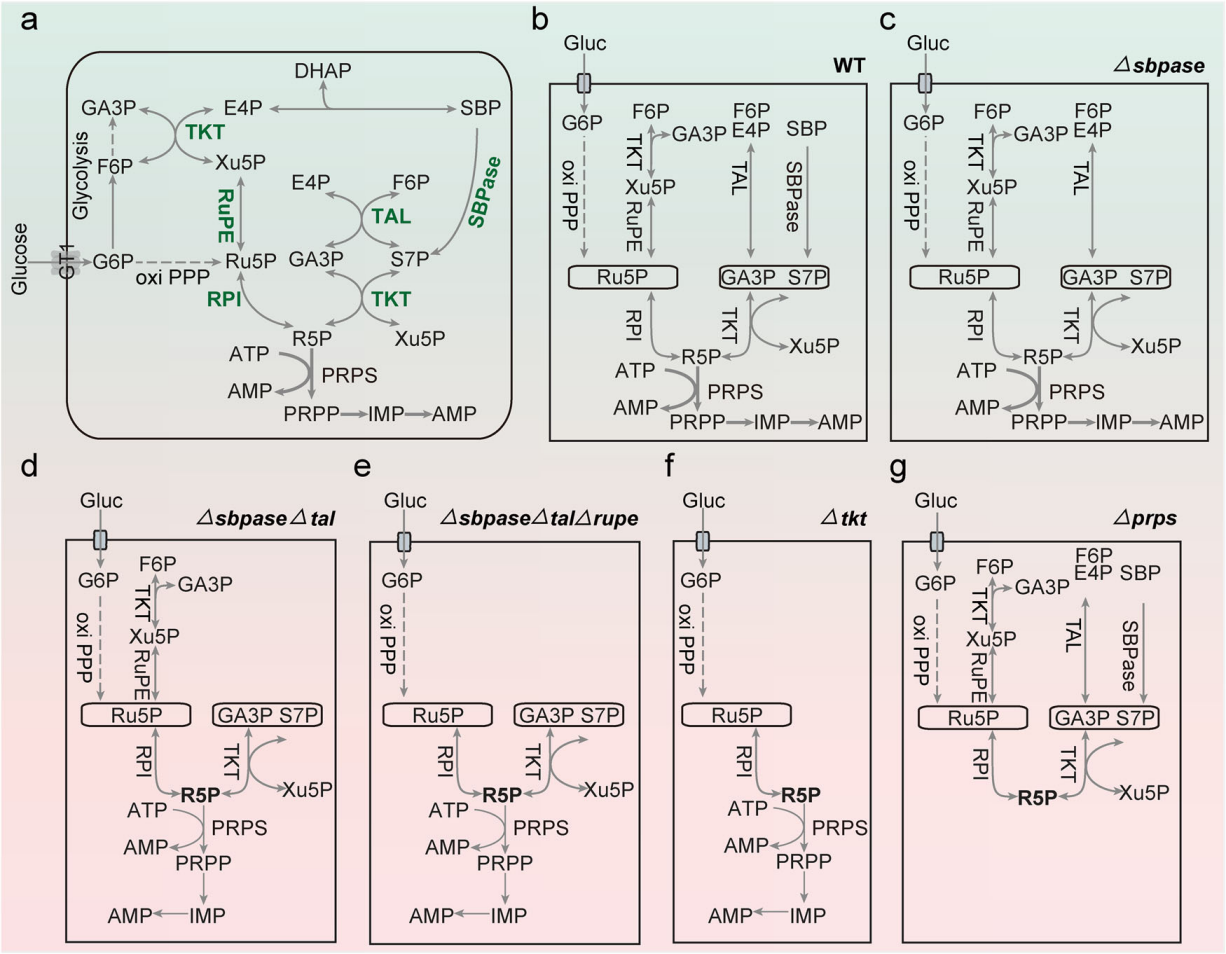
Ribose-5-phosphate synthesis and utilization in *Toxoplasma* in disparate environments. **a**, **b** Potential routes of ribose-5-phosphate (R5P) synthesis—SBPase*-*TKT, TAL-TKT, TKT-RuPE-RPI and oxiPPP-RPI pathways. R5P produced via PPP is a substrate for phosphoribosyl-pyrophosphate synthetase (PRPS) to make PRPP, a crucial precursor for nucleotide biogenesis. **c** *SBPase* disruption enables the R5P synthesis through other routes. **d** The *ΔsbpaseΔtal* strain meets its need for R5P via the TKT-RuPE-RPI, oxiPPP-RPI and TKT-mediated pathways. However, None of these routes appear to be adequate for parasite growth. **e** The supply of R5P depends on the oxiPPP-RPI and TKT-mediated pathways in the *ΔsbpaseΔtalΔrupe* strain. **f** TKT cooperates with SBPase, TAL and RuPE-RPI pathways to satisfy R5P requirements. Mutants lacking *TKT* rely only on the oxiPPP-RPI pathway for supplying R5P, which may not be sufficient. **g** Deletion of *PRPS* blocks the biosynthesis of PRPP and parasite growth, even though PPP can provide R5P.

Metabolic pathways required for the lytic cycle of *T. gondii* are promising drug targets for therapeutic intervention^14–22^. The pentose phosphate pathway (PPP) is the primary route for making ribose-5-phosphate, an essential precursor for nucleic acid synthesis. *Toxoplasma* possesses a functional PPP, divided into oxidative and nonoxidative branches and localized in the cytoplasm and nucleus^6^. Our previous work showed the importance of oxidative PPP in tachyzoites of *T. gondii*^6^. Herein, we demonstrate the physiological, functional and therapeutic relevance of the nonoxidative PPP (Fig. 8).

Our data suggest that TKT, in alliance with TAL and SBPase, allows the parasite to reprogram its carbon flux to R5P in varying nutritional environments (Fig. 8a, b). As shown, a loss of SBPase would impair the production of S7P from SBP; however, it could be effectively supplied through the canonical PPP, rendering SBPase nonessential in tachyzoites (Fig. 8c). In a previous report, the deletion of SBPase has bee shown to be modestly detrimental to the parasite growth^10^. We speculate that the RH*Δku80Δsbpase* or *DiCreΔsbpase* mutants exhibit an adaptive compensation by other pathways. The deletion of *SBPase* also does not notably affect yeast growth, although it drives SBP flux into the nonoxidative PPP^8^. Our preceding study and this work, found that the deletion of *TAL*, expressed in the parasite nucleus, did not impact the lytic cycle^6^. Ironically, even deletion of both *SBPase* and *TAL* inflicts only a mild phenotypic defect in tachyzoites, possibly because they can depend on the TKT-RuPE-RPI and oxidative PPP-RPI pathways for their survival (Fig. 8d). In procyclic *T. brucei*, *TKT* deletion does not cause apparent morphological change or growth defect^23,24^. On the other hand, the *TKT*-null yeast mutant exhibits impaired growth on fermentable but not on gluconeogenic substrates^25^. Surprisingly, TKT is crucial for the development of *T. gondii* tachyzoites. We postulate that although R5P produced by the oxidative PPP-RPI pathway can support parasite survival in the absence of *TKT*, it is insufficient to sustain optimal growth (Fig. 8f). Moreover, PRPS catalysis of R5P into PRPP is critical for tachyzoites. The R5P production remains intact after *PRPS* deletion, but the metabolite cannot be utilized for downstream biosynthesis, resulting in parasite death (Fig. 8g).

TKT can catalyze the formation of E4P and GA3P from F6P and Xu5P, and E4P is a crucial precursor of biosynthesis of aromatic amino acids such as tyrosine, tryptophan and phenylalanine^25–27^. Hence, *TKT*-deficient parasites should be strictly auxotrophic for these amino acids. Previous studies have shown that the *Tg*ApiAT5-3 transporter can import tyrosine into *T. gondii*^28^. Although transporters for tryptophan and phenylalanine have not been identified in the apicomplexan parasites, the stable-isotope labeling has shown that intracellular tachyzoites can salvage phenylalanine from the host^29,30^. Equally, *T. gondii* is long known to depend on host-derived tryptophan^30,31^. It is, therefore, plausible that TKT does not play a primary role in the biogenesis of aromatic amino acids. On a different note, TKT can bridge the PPP and glycolysis by sharing F6P and GA3P. Upon its disruption, a significant pool of carbon is trapped as oxidative PPP metabolites, leading to an imbalance of PPP, glycolysis, and accumulation of toxic metabolites. In particular, the accrual of 6-phosphogluconate and fructose-1,6-bisphosphate (FBP) is harmful in other systems^25,32–34^. Consistent with this hypothesis, a previous study on *T. gondii* tachyzoites showed that the ALD mutant was strongly inhibited glucose-containing medium, likely due to toxicity resulting from FBP accumulation^35^.

Our proteomics identified a repertoire of dysregulated proteins in the *Δtkt* strain. A substantial decline in proteins of nucleotide biogenesis in the mutant is probably a direct consequence of disrupted R5P synthesis upon loss of *TKT*. We also observed multiple rhoptry and microneme proteins repressed in the *Δtkt* mutant. Among rhoptry proteins, ROP1 is vital for *T. gondii* to resist interferon gamma-mediated innate immune response, and its deletion profoundly affects the virulence of PRU strain in C57BL/6 J mice^36^. Similarly, ROP18 is a highly polymorphic protein kinase that determines the virulence of types I and II strains of *T. gondii*^37^. In addition, ROP39 is an Irgb10-specific effector contributing to the tachyzoite virulence^38^. Of the perturbed micronemal proteins, the MIC1/MIC4/MIC6 complex plays a vital role in host-cell attachment, invasion, pathogenesis and immune evasion^39–41^. Correspondingly, the invasion efficiency of *Δmic1* and *Δmic6* strains is significantly reduced^39,40^. The expression of ROP1, ROP18, ROP39, MIC1, MIC4 and MIC6 was markedly downregulated in the *TKT*-knockout parasites, consistent with the phenotypic data.

The protective efficacy and safety of the *Δtkt* mutant requires further analysis in food-producing animals and the final host (cats). Previous studies have shown that deletion or knockdown of other proteins, such as carbamoyl phosphate synthetase II (CPSII), orotidine-5′-monophosphate decarboxylase (OMPDC), lactate dehydrogenase 1 (LDH1), phosphatidylthreonine synthase (PTS), CDP-DAG synthase (CDS) and ethylene inducible protein (PDX1) results in “metabolically-attenuated vaccines”^14,21,42–46^. Therefore, disrupting the *CPSII*, *OMPDC*, *LDH1, PTS, CDS* or *PDX1* in the *Δtkt* mutant could further attenuate the *T. gondii* virulence and improve the safety of live vaccine strains. Our work underpins the importance of nonoxidative PPP during the lytic cycle of *T. gondii*. It consolidates that R5P is essential for tachyzoite growth and survival despite the redundancy in its biogenesis. Moreover, we reveal TKT and PRPS as potential therapeutic drug targets.

## Methods

### Animal studies and ethical approval

Seven-week-old female ICR mice were purchased from the Guangdong Medical Experimental Animal Center (Guangdong Province), while Balb/c-nu mice were acquired from Guangzhou Ruige Biotechnology Co., LTD. Mice were hosted in clean filter-top cages with a 12:12 light-dark cycle, 50–60% humidity, and room temperature (22 °C) following standard protocols as the regulations of the Administration of Affairs Concerning Experimental Animals (Permit 2022f210, the Ethical Committee of South China Agricultural University, Guangzhou)

### Biological resources

The *Δ6pgdh2* and *Δrpi* mutants were generated in our previous work^6^. Cell culture media and additives were purchased from Gibco (Rockville, MD, USA). Other common chemicals were procured from Sigma-Aldrich (Missouri, USA). 1,2-^13^C_2_ glucose was received from Sigma-Aldrich, and fluorophore-conjugated antibodies (Alexa488/594, 1:1000) were obtained from Cell Signaling Technology (Massachusetts, USA). Dolichos Biflorus Agglutinin (DBA, 1:100) was purchased from Vector Laboratories (San Francisco, USA). The ATP assay kit was acquired from Beyotime (Shanghai, China).

### Plasmid construction, parasite culture and transfection

Primers (Supplementary Data 1) were synthesized by Sangon (Sangon Biotech, Shanghai, China). Locus-specific CRISPR/Cas9 and other plasmids (Supplementary Data 2) were constructed by multi-fragment ligation using the one-step cloning kit (ClonExpress II, Vazyme Biotech Co., Ltd, Nanjing). All plasmids were verified by DNA sequencing before use. Type I (RH*Δku80*, RH*-Luc* and *DiCre*^47^), type II (ME49, PRU) and type III (VEG) tachyzoites (parental and derivative strains listed in Supplementary Data 3) were grown in human foreskin fibroblast (HFF) cells (ATCC, USA) maintained in Dulbecco’s Modified Eagle’s Medium (DMEM, Gibco Life Technologies, Rockville, MD, USA) supplemented with 2% fetal bovine serum (FBS), glutamine (2 mM), penicillin (10 U/mL) and streptomycin (100 μg/mL). The locus-specific CRISPR/Cas9 plasmids and corresponding homologous donor templates (listed in Supplementary Data 1 and 2) were transfected in extracellular tachyzoites. Transgenic parasites were selected by 1 μM pyrimethamine (Sigma-Aldrich, Missouri, USA) and cloned by limiting dilution. The *DiCreTgTKT-Ty* was engineered by co-transfecting a *UPRT*-specific CRISPR construct and a TKT-expressing cassette into the *DiCre* strain, followed by selection with 10 μM FUdR. The comp*SBPase* and comp*TAL* strains were constructed by inserting a *Tg*SBPase or *Tg*TAL expression cassette at the *UPRT* locus of the *ΔsbpaseΔtal* strain and selected with FUdR. To generate the *ΔsbpaseΔtalΔhd*, *ΔsbpaseΔtalΔsrs12d*, and *ΔsbpaseΔtalΔrupe* strains, we treated the *ΔsbpaseΔtal* mutants with rapamycin for 2 days to excise the loxp-DHFR*-loxp. In the next step, we transfected the *HD*, *SRS12D*, or *RuPE*-specific CRISPR plasmids along with respective homology donor amplicons, into pyrimethamine-sensitive *ΔsbpaseΔtal* tachyzoites, followed by drug selection.

### Protein expression, antibody production and immunofluorescence assays

The full-length coding sequences of *TgSBPase*, *TgTAL* and *TgTKT* were amplified from the cDNA of the RH*Δku80* strain and cloned into the *pCold* vector containing a 6×His tag. The epitope-tagged proteins were expressed in *E. coli* BL21 (DE3) cells and analyzed for expression by SDS-PAGE analyses. Subsequently, 7-week-old ICR mice were subcutaneously immunized with the purified recombinant proteins, and the antiserum was collected to localize the native proteins in *T. gondii*. Immunofluorescence imaging was executed as described elsewhere^48^. Briefly, the RH*Δku80* tachyzoites were used to infect confluent HFF monolayers on glass coverslips. Samples were fixed with 4% paraformaldehyde, permeabilized in 0.1% Triton X-100 for 20 min and blocked overnight with 10% FBS. Parasitized host cells were treated with appropriate dilutions of rabbit anti-*Tg*ALD antibody (a cytoplasm marker, 1:1000), and either mouse anti-*Tg*SBPase, anti-*Tg*TAL or anti-*Tg*TKT serum (60 min at 37 °C, 1:1000), washed with PBS and then stained with Alexa Fluor 594 (goat anti-rabbit IgG), Alexa Fluor 488 (goat anti-mouse IgG) antibodies and Hoechst (a nucleus marker, 1:2000). Cells embedded in fluorescein were visualized on a BX53 microscope (Olympus, Japan).

### Lytic cycle assays

Confluent monolayers of HFFs in 6-well plates were infected with 100 freshly-egressed parasites per well and incubated without perturbation at 37 °C with 5% CO_2_ for 7 or 14 days, as reported earlier^49^. The infected cells were fixed with 4% paraformaldehyde, and the parasite plaques were visualized by crystal violet (0.1%). The plaque size and number were scored after scanning 6-well plates (Microtek Scan Marker i600, MICROTEK, China). For replication, the *TgTKT-TyΔtkt* strains pretreated with rapamycin for 4 days were allowed to infect fresh HFFs on coverslips for 2 h. *TgPRPS-cKD* tachyzoites were pretreated with rapamycin for 5 days, and YFP^+^*Tg*PRPS^-^ strains were purified by fluorescence sorting. Subsequently, HFFs on coverslips were infected by tachyzoites of the RH*Δ*ku80, *Δsbpase*, *DiCre, DiCre-YFP*^+^, *DiCreΔsbpase*, *ΔsbpaseΔtal*, *ΔsbpaseΔtalΔhd*, *ΔsbpaseΔtalΔrupe*, *ΔsbpaseΔtalΔsrs12d*, comp*SBPase*, comp*TAL*, YFP^+^*Tg*PRPS^-^ and *Tg*PRPS-*cKD* strains for 1–2 h. Extracellular parasites were washed away by PBS, and intracellular ones were cultured for an additional 24 h.

Samples were immunostained by rabbit anti-*Tg*ALD (before permeabilization) and mouse anti-*Tg* IgG (after permeabilization, 1:1000) to visualize non-invaded and total parasites, respectively. The *DiCre-YFP*^*+*^, *TgTKT-TyΔtkt* and *Tg*PRPS-*cKD* strains pretreated with rapamycin were visualized by YFP-positive signal and rabbit anti-*Tg*ALD (before permeabilization). The *ΔsbpaseΔtalΔhd*, *ΔsbpaseΔtalΔrupe*, and *ΔsbpaseΔtalΔsrs12d* strains were immunostained only using the rabbit anti-*Tg*ALD antibody owing to their YFP^+^ signal. The number of parasites per vacuole was counted in multiple random fields using a BX53 microscope (Olympus, Japan). A minimum of 150 vacuoles were examined for each sample.

For invasion assay, HFF cells were infected (2 × 10^5^ parasites/strain, MOI = 1:3) and incubated for 30 min at 37 °C with 5% CO_2_. After PBS washing, samples were fixed by 4% paraformaldehyde, incubated with rabbit anti-*Tg*ALD for 20 min, permeabilized in 0.1% Triton X-100 for 15 min, and blocked with 10% FBS for 2 h. The *DiCre* strain was incubated with mouse anti-*Tg*IgG for 20 min, while this step was omitted for the YFP-positive *Δtkt* mutant. The *DiCre* strain was stained with Hoechst, goat anti-mouse lgG and anti-rabbit lgG secondary antibodies, while the *Δtkt* strain was stained with Hoechst and goat anti-rabbit lgG. The parasites invading host cells were counted under a fluorescence microscope, and at least 150 fields were scored for each sample.

### Morphometric analysis of parasites

The morphometric assay was performed, as reported elsewhere^50^. Briefly, intracellular parasites were stained with the appropriate primary (mouse anti-*Tg*GAP45, 1:1000; rabbit anti-*Tg*ALD) and secondary (Alexa Fluor 488, Alexa Fluor 594) antibodies for 1 h each. Samples were washed with PBS between different treatments. The length and width of *Tg*GAP45-labeled parasites were measured using standard cellSens software (OLYMPUS).

### Virulence test and parasite burden in mice

The female ICR mice (7-week-old) were infected with 100 tachyzoites of the RH*Δku80*, *Δsbpase, DiCre*, *ΔsbpaseΔtal*, *ΔsbpaseΔtalΔhd*, *ΔsbpaseΔtalΔsrs12d*, *DiCre-YFP*^*+*^and *Δtkt* strains by intraperitoneal injection (8, 10 or 11 mice in each group), as specified in figure legends. Infection by the *ΔsbpaseΔtalΔrupe* mutant was performed at doses of 10^4^ and 10^5^ parasites (5 mice in each group), and the *Δtkt* mutant was inoculated at doses of 10^3^, 10^4^,10^5^, 10^6^ (8 mice in each group) and 10^7^ (5 mice) parasites. The clinical signs and survival of mice were monitored daily. Blood samples collected from surviving animals were tested 21 or 30 days post-infection, and mice seronegative by immunofluorescence were excluded from further analysis. The ICR or Balb/c-nu mice were infected with 10^4^ parasites, and then the parasite burden was measured 5 days post-infection. The genomic DNA from the peritoneal fluid, liver, spleen and ileum was extracted using the TIANamp Blood DNA Kit (Tiangen Biotech Co. Ltd, Beijing, China) and subjected to qPCR, as described previously^44,51^.

### Immune protection and bioluminescence imaging

Animals surviving the primary infection with a dose of 10^2^, 10^3^ or 10^4^ *Δtkt* parasites were challenged by type I (*DiCre*), type II (ME49 and PRU) or type III (VEG), respectively (5 mice/group, 10^4^ tachyzoites/mouse). The naïve mice inoculated with the same dose of indicated strains served as the control group (5 or 10 animals/group). The clinical signs and survival of mice were monitored daily for 21 or 30 days, and the cumulative mortality was analyzed (GraphPad Software Inc., La Jolla, CA, USA). To examine whether the “*Δtkt* vaccination” could block cyst formation upon challenge infection, the female ICR mice were infected with the *Δtkt* parasites (10^4^). After 21 days of inoculation, the “*Δtkt*-vaccinated” and naïve animals were challenged with 10^4^ tachyzoites (intraperitoneal injection) of the PRU and VEG strain. They were monitored for another 21 days to assess clinical signs and survival. Seropositive mice were sacrificed at the end of the immunoprotection test, and their brains were collected. The number of tissue cysts in each brain sample was determined as described previously^44,45^. For imaging, naïve or *Δtkt-*immunized animals were infected by a luciferase-expressing RH-*Luc* strain (5 mice/group, 10^4^ parasites/mouse, i.p.). Animals were anesthetized five days post-infection using 2% isoflurane and injected with 300 μL of 15 mg/ml D-Luciferin (Yeasen Biotechnology Co., Ltd, Shanghai, China), as reported^45^. The in vivo growth of RH-*Luc* strains was monitored by bioluminescence imaging on an IVIS Spectrum imaging system (PerkinElmer, Inc., Boston, MA, USA).

### Growth analysis and competition assay

Confluent HFF cells in 12-well plates were infected by the *DiCre*, *Δ6pgdh2*, *Δrpi*, *Δtkt* strains (1 × 10^3^ tachyzoites/well, 37 °C with 5% CO_2_). The parasites were collected on days 0, 1, 2, 3, 4, 5 and 6 and analyzed by qPCR to determine the growth (primers in Supplementary Data 1). For the competition assay, the *TgPRPS*-*cKD* tachyzoites were cultured without or with rapamycin for 5 days. Afterward, YFP^-^*Tg*PRPS^+^ and YFP^+^*Tg*PRPS^−^ parasites were mixed (ration 1:1), and HFFs were infected. The proportions of YFP^+^ and YFP^-^ parasites in each passage were determined by flow cytometry. After each passage, tachyzoites (1 mL culture) were collected for analysis on a CytoFLEX (10,000 events/sample, Beckman Coulter, Inc., USA).

### Bradyzoite differentiation and amylopectin staining

The *DiCre* and *Δtkt* strains cultured under standard culture conditions were used to infect HFFs seeded on glass coverslips. Parasitized cells were fixed, permeabilized, blocked and stained for 2 h with Dolichos Biflorus Agglutinin (Vector Laboratories, California) and 30 min with the Hoechst dye. A minimum of 100 vacuoles containing 4 or more parasites were examined for DBA signal. To stain amylopectin granules, host cells harboring the *DiCre* and *Δtkt* strains were incubated with Hoechst and subsequently with periodic acid solution (5 min, room temperature) reagents as described previously^52^. After washing with PBS, samples were incubated with Schiff’s reagent for 15 min. Finally, the hematoxylin solution was used to stain cells for 5 min, followed by PBS washing. The PAS-positive parasites were visualized by a BX53 microscope.

### Semi-quantitative RT-PCR and quantitative real-time PCR

The *DiCre*, *ΔsbpaseΔtal* and *Δtkt* parasites were filtered through 3 μm polycarbonate membranes and harvested by centrifugation. Total RNA was extracted (Promega Biotech Co. Ltd, Beijing) and reverse-transcribed to cDNA (Yeasen Biotechnology Co. Ltd., Shanghai). Semi-quantitative PCR was executed to validate the gene knockout using the equivalent cDNA of *Dicre*, *ΔsbpaseΔtal*, and *Δtkt* strains. Quantitative real-time PCR of *Tg*BT1, *Tg*HD, *Tg*SRS12D, *Tg*MIC4, *Tg*MIC6, *Tg*ROP39, *Tg*SRS44, *Tg*Rab2 and β-tubulin (reference) transcripts in the parental and transgenic stains was performed using SYBR Green PCR mix (TOYOBO, Osaka, Japan). Primers for the semi-quantitative RT-PCR and real-time PCR are listed in Supplementary Data 1.

### Whole genome sequencing

Freshly egressed *ΔsbpaseΔtal* tachyzoites were purified using a 3 μm polycarbonate membrane filter and washed with PBS. Pellets containing approximately 10^8^ tachyzoites were lysed in the GB buffer, and DNA was prepared using the TIANamp Blood DNA Kit (Tiangen Biotech Co. Ltd, Beijing). The purified genomic DNA was sequenced on Illumina NovaSeq6000. Subsequently, the clean reads were mapped to the reference genome of the *Toxoplasma* GT1 strain. The mapping results were visualized by an integrative genomics viewer program.

### RNA sequencing

Total RNA of the *DiCre* and *ΔsbpΔtal* tachyzoites isolated using Transzol UP Reagent (TransGen Biotech Co., Ltd, Beijing, China) was assessed for the quality and quantity using a NanoPhotometer (Thermo Fisher Scientific, MA, USA) and 2100 RNA Nano 6000 Assay Kit (Agilent Technologies, CA, USA). Subsequently, RNA sequencing was performed, as reported^53^. The RNA-seq library was sequenced using the Illumina NovaSeq 6000 sequencer. SeqPrep (https://github.com/jstjohn/SeqPrep) and Sickle (https://github.com/najoshi/sickle) were deployed to remove low-quality reads. The clean reads were aligned to the genome of the GT1 strain by using HISAT2 software. The expression level of transcripts was quantified and normalized using the TPM (transcripts per million) to identify differentially expressed genes between two groups. The GO and KEGG programs were deployed for the functional enrichment analyses.

### Metabolomics (UPLC-MS)

The intracellular *ΔsbpaseΔtal*, *Δtkt* and *DiCre* strains were cultured (12 h) in DMEM containing 8 mM 1,2-^13^C_2_-glucose (Sigma-Aldrich, Missouri, USA). 5 × 10^7^ parasites were used for metabolite extraction, as described elsewhere^6^. To determine the role of TKT, the extracellular *DiCre* and *Δtkt* parasites (3 × 10^7^) were labeled (4 h) with 8 mM 1,2-^13^C_2_-glucose in DMEM. Subsequently, the parasites were washed with PBS, pelleted and extracted in 50% aqueous acetonitrile. Metabolites were analyzed by UHPLC-MS, as reported earlier^6^. To examine the abundance of pentose sugars and nucleotides, the *Δtkt* (5 × 10^7^) and *ΔsbpaseΔtal* (3 × 10^7^) parasites were subjected to metabolite extraction for UHPLC-HRMS analysis^6^. The control group consisted of the same amount of parental *DiCre* parasites. The target peaks were quantified by the Xcalibur software (version 3.0.63, Thermo Fisher Scientific)^54^, and the relative abundances of pentose sugars and nucleotides were obtained using the added phenylalanine-d5 as a reference.

### ATP quantification

Fresh extracellular parasites (5×10^6^ cells/strain) were lysed in 200 μL lysis buffer on ice for 30 min and centrifuged at 12,000 g for 5 min. The ATP levels in the supernatant were detected using a commercial kit (Beyotime, Shanghai, China), as described previously^55,56^. Briefly, 100 μL of each sample or serially-diluted ATP standard solution (3 μM, 1 μM, 0.3 μM, 0.1 μM, 0.03 μM, 0.01 μM and 0 μM) was mixed with 100 μL of ATP detection solution in opaque 96-well plates. The bioluminescence signal in each well was determined by a multi-mode plate reader (Synergy, BioTek Instruments, USA).

### Proteomics

A total of 3 × 10^7^ *DiCre-YFP*^*+*^ and *Δtkt* parasites were lysed (1% SDS,1% protease inhibitor cocktail) using an ultrasonic processor and mixed with a five-fold excess of cold acetone (−20 °C, 2 h). Insoluble material was pelleted (4500 g, 5 min, 4 °C), washed 2× with cold acetone (Zhejiang Hannuo Chemical Technolog., Zhejiang, China) and redissolved in 200 mM tetraethylammonium bromide (Sigma-Aldrich, Missouri, USA). Proteins were then hydrolyzed overnight with trypsin (1:50). The next day, the protein solution was reduced at 56 °C with 5 mM DL-dithiothreitol (Sigma-Aldrich, Missouri, USA) for 30 min and alkylated with 11 mM iodoacetamide (Sigma-Aldrich, Missouri, USA) in the dark for 15 min at room temperature. Samples were analyzed, as reported elsewhere^6^.

### Data plotting and statistical analysis

All experiments were performed three independent times unless stated otherwise. Statistical analyses were executed in GraphPad Prism 8.0.1 (La Jolla, CA, USA) using Student’s *t*-tests, log rank Mantel–Cox test and two-way ANOVA, as indicated in the figure legends.

### Reporting summary

Further information on research design is available in the Nature Portfolio Reporting Summary linked to this article.

### Supplementary information

Supplementary information

Peer Review File

Description of Additional Supplementary Files

Supplememtary Data 1

Supplememtary Data 2

Supplememtary Data 3

Supplememtary Data 4

Supplememtary Data 5

Supplememtary Data 6

Supplememtary Data 7

Supplememtary Data 8

Supplememtary Data 9

Supplememtary Data 10

Reporting Summary

### Source data

Source data

## Data Availability

The genome sequencing data have been deposited and released with the accession number PRJNA947326. Transcriptomics sequencing data are available in the short read archive (SRA) of the National Center for Biotechnology Information database under the accession number PRJNA947324. The raw proteomics data have been deposited in the ProteomeXchange Consortium (www.proteomexchange.org) via the iProX partner repository with the accession number PXD041014. All other data are presented in the article or the supplementary information. Reference genome of the *Toxoplasma* GT1 strain: https://toxodb.org/toxo/app/record/dataset/NCBITAXON_507601. Source data are provided with this paper.
