# Supplementary material for: Metabolic plasticity, essentiality and therapeutic potential of ribose-5-phosphate synthesis in Toxoplasma gondii
Source: Nat Commun. 2024 Apr 8;15:2999. doi: 10.1038/s41467-024-47097-8 (PMC11001932; doi:10.1038/s41467-024-47097-8)
Supplement: Supplementary file 1 — Supplementary information [file 41467_2024_47097_MOESM1_ESM.pdf]

Supplementary Information for

Metabolic plasticity, essentiality and therapeutic potential of ribose-5-phosphate synthesis in *Toxoplasma gondii*

Xuefang Guo [1,2,#], Nuo Ji [1,2,#], Qinghong Guo [1,2], Mengting Wang [1,2], Huiyu Du [1,2], Jiajia Pan [1,2], Lihua Xiao [1,2], Nishith Gupta [3,4,*], Yaoyu Feng [1,2,*], Ningbo Xia [1,2,*]

[1] State Key Laboratory for Animal Disease Control and Prevention, South China Agricultural University, Guangzhou, China
[2] Guangdong Laboratory for Lingnan Modern Agriculture, Center for Emerging and Zoonotic Diseases, College of Veterinary Medicine, South China Agricultural University, Guangzhou, Guangdong, China
[3] Intracellular Parasite Education and Research Labs (iPEARL), Department of Biological Sciences, Birla Institute of Technology and Science, Pilani (BITS-P), Hyderabad, India
[4] Department of Molecular Parasitology, Faculty of Life Sciences, Humboldt University, Berlin, Germany

[#] These authors contributed equally to this work.

[*] Corresponding author: Nishith Gupta, Yaoyu Feng, Ningbo Xia

Email:
Gupta.Nishith@hyderabad.bits-pilani.ac.in;
yyfeng@scau.edu.cn;
ningboxia@scau.edu.cn

Running title: Ribose-5-phosphate Synthesis and Utilization in *Toxoplasma*

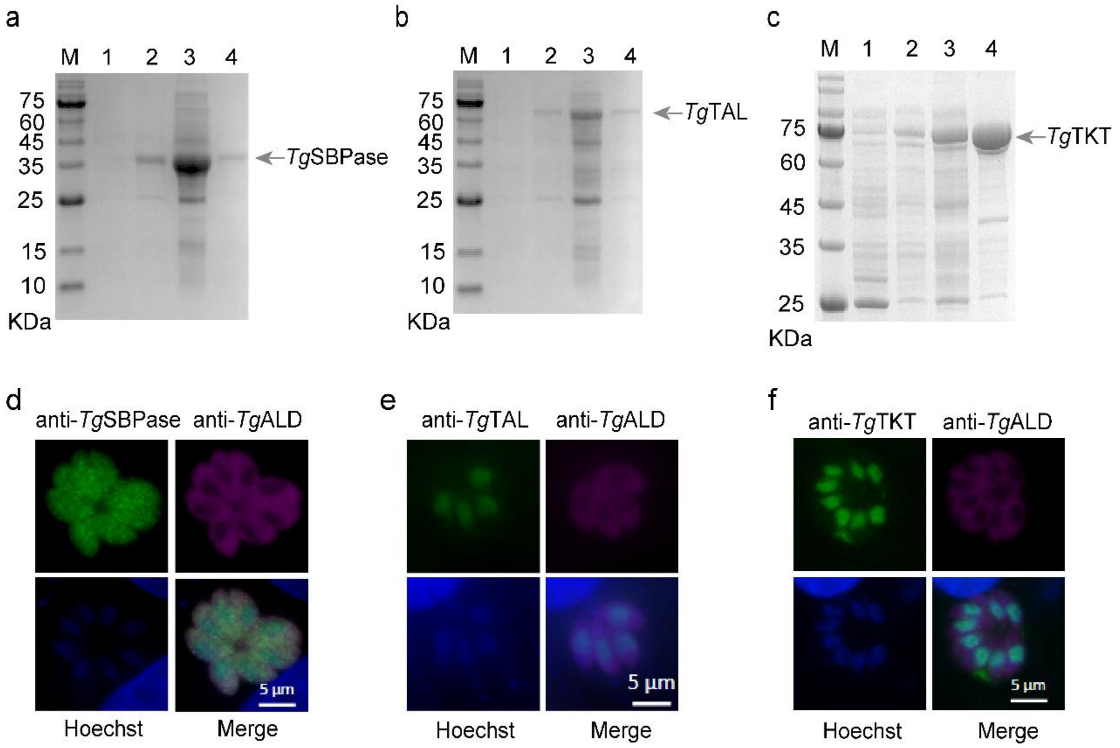

Supplementary Fig. 1: Expression of recombinant SBPase, TAL and TKT in *E. coli* and localization in *T. gondii*.

a-c SDS-PAGE analysis of indicated proteins. M: molecular weight markers; Lane 1: lysate from recombinant bacteria without IPTG induction; Lane 2: lysate from IPTG-induced bacteria; Lane 3: Supernatant of 6×His-protein; Lane 4: Inclusion body of 6xHis-protein. **d-e** Subcellular localization of the native SBPase, TAL and TKT proteins. ALD and Hoechst were used as the cytoplasmic and nucleus markers, respectively. Scale bars = 5 µm. Source data are provided as a Source data file.

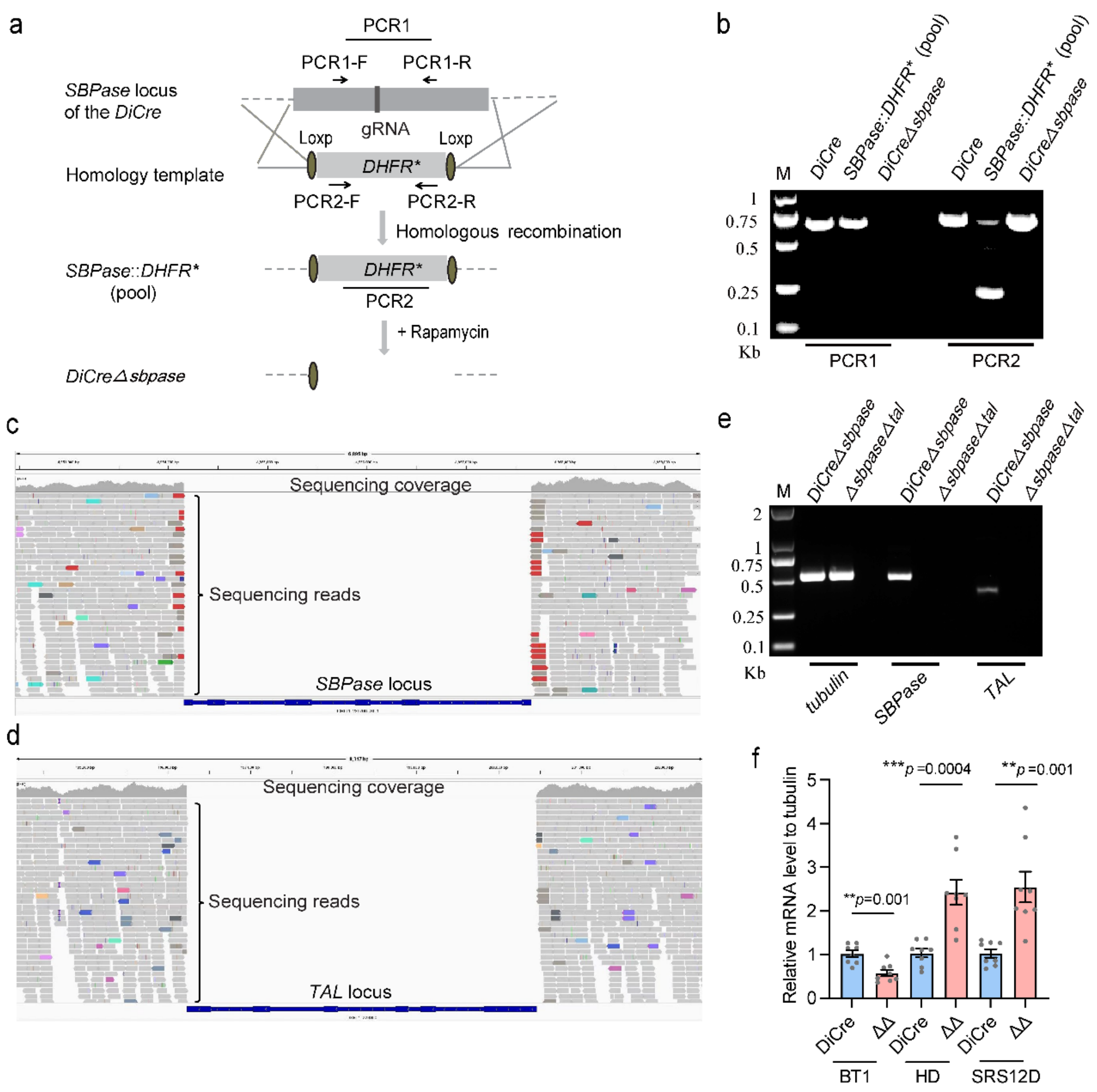

Supplementary Fig. 2: Engineering of the *SBPase* mutant and characterization of the *SBPase-TAL* double deletion strain.

a Schematics showing the construction of the *DiCreΔsbpase* strains. **b** Diagnostic PCRs confirming the *DiCreΔsbpase* mutant. **c-d** Sequencing of the *ΔsbpaseΔtal* double mutant. Genomic DNA was subjected to sequencing, and clean reads of the double mutant genome were aligned to the reference GT1 strain and visualized using Integrative Genomics Viewer 2.16.2. **e** Semi-quantitative RT-PCR confirming the absence of *SBPase* and *TAL* expression in the *ΔsbpaseΔtal* mutant (β-tubulin, control transcript). **f** Quantitative RT-PCR validation of the indicated transcripts. β-tubulin was used as an internal reference (eight replicates in total from two independent experiments, means ± SEM; unpaired two-tailed Student's *t*-test). Source data are provided as a Source data file.

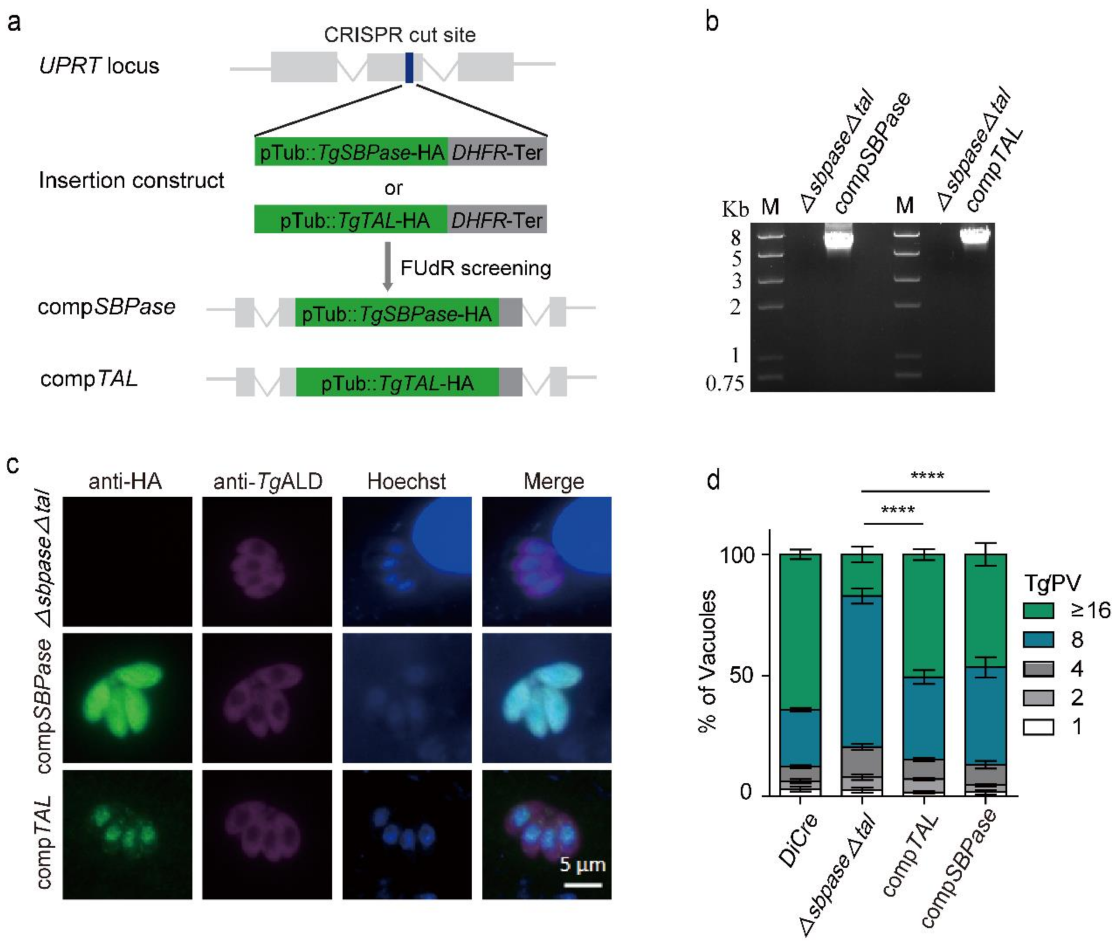

Supplementary Fig. 3: Construction of the SBPase and TAL complementation strains.

a Illustration for inserting the SBPase-HA or TAL-HA expression cassette at the *UPRT* locus of the *ΔsbpaseΔtal* mutant to generate the corresponding complementation strains (comp*SBPase* and comp*TAL*). **b** Genomic PCR to decipher the integration of SBPase or TAL-expressing cassette at the *UPRT* locus. **c** Expression of SBPase and TAL in the comp*SBPase* and comp*TAL* strains, respectively. The comp*SBPas*e and comp*TAL* strains were stained with mouse anti-HA and rabbit anti-*Tg*ALD antibodies. Scale bars = 5 µm. **d** Replication rates of the indicated strains. The data show means ± SEM from four assays, each with two replicates (****, $p <$ 0.0001, two-way ANOVA). Source data are provided as a Source data file.

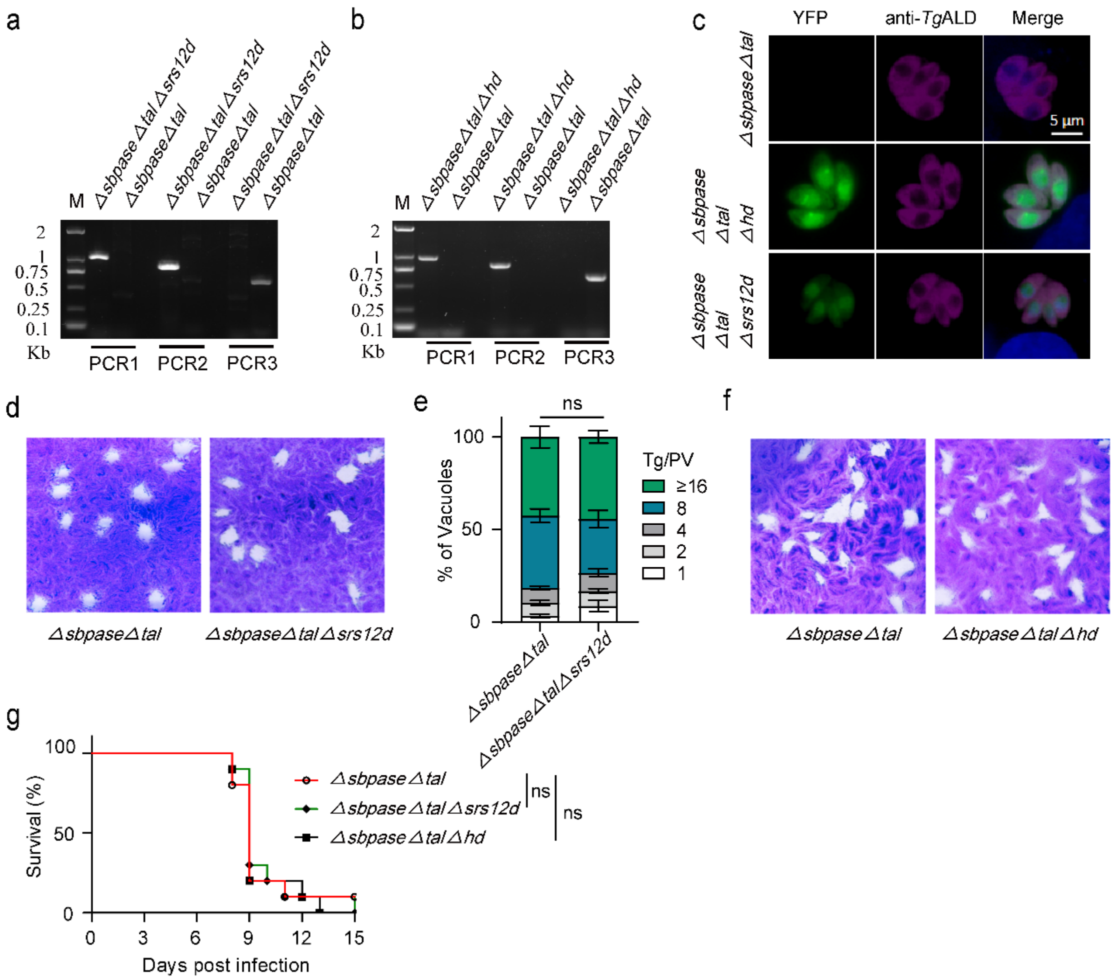

Supplementary Fig. 4: Construction and characterization of the *ΔsbpaseΔtalΔsrs12d* and *ΔsbpaseΔtalΔhd* strains.

a-b Diagnostic PCRs confirming the *ΔsbpaseΔtalΔsrs12d* and *ΔsbpaseΔtalΔhd* mutants. **c** Immunofluorescence staining revealing the disruption of *SRS12D* and *HD* through the insertion of a YFP-selectable marker. Scale bars = 5 μm. **d** Plaque assay comparing the *ΔsbpaseΔtalΔsrs12d* and *ΔsbpaseΔtal* strains (7 days). **e** Replication rate of the *ΔsbpaseΔtalΔsrs12d* and *ΔsbpaseΔtal* mutants (n = 3 assays, means ± SEM; ns, not significant, $p = 0.1668$, two-way ANOVA). **f** Plaque assay performed with the *ΔsbpaseΔtalΔhd* and *ΔsbpaseΔtal* strains. **g** Survival curves of mice infected by the *ΔsbpaseΔtal*, *ΔsbpaseΔtalΔsrs12d* or *ΔsbpaseΔtalΔhd* strains (10 mice/strain). Statistical significance tested by log rank Mantel-Cox test; ns, not significant, $p = 0.9759$ (*ΔsbpaseΔtal vs ΔsbpaseΔtalΔsrs12d*), $p = 0.9737$ (*ΔsbpaseΔtal vs ΔsbpaseΔtalΔhd*). Source data are provided as a Source data file.

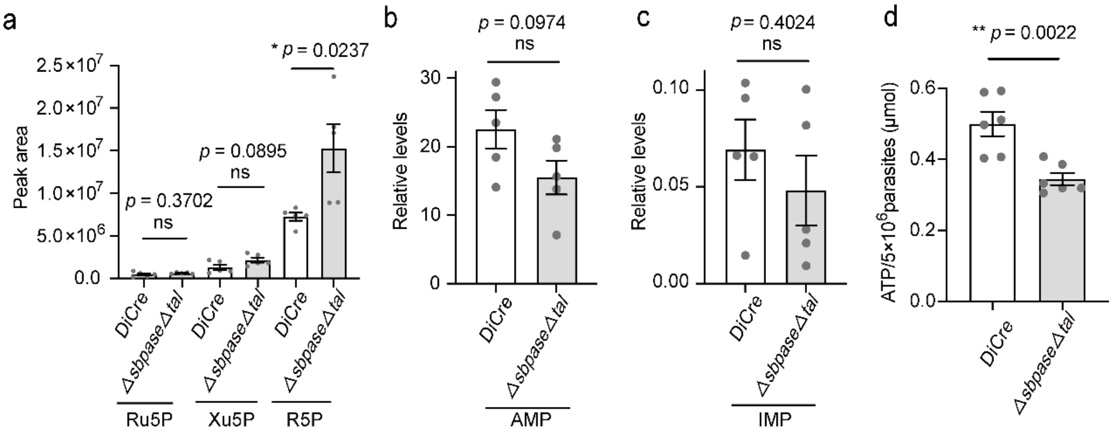

Supplementary Fig. 5: Collective loss of *SBPase* and *TAL* is associated with reduced ATP content and accumulation of pentose sugars.

a Quantification of Ru5P, Xu5P and R5P. Our metabolomic analysis shows cumulative unlabelled and labeled peaks (peak area ± SEM, n = 5 experiments, ns, not significant, unpaired two-tailed Student's *t*-test). **b-c** Relative levels of AMP and IMP in intracellular *DiCre* and *ΔsbpaseΔtal* parasites. UHPLC-HRMS was used to analyze AMP and IMP (n = 5 experiments, means ± SEM; ns, not significant, unpaired two-tailed Student's *t*-test). **d** The ATP levels in the parental (*DiCre*) and *ΔsbpaseΔtal* tachyzoites (means ± SEM; n = 3 experiments, each with two replicates, unpaired two-tailed Student's *t*-test). Source data are provided as a Source data file.

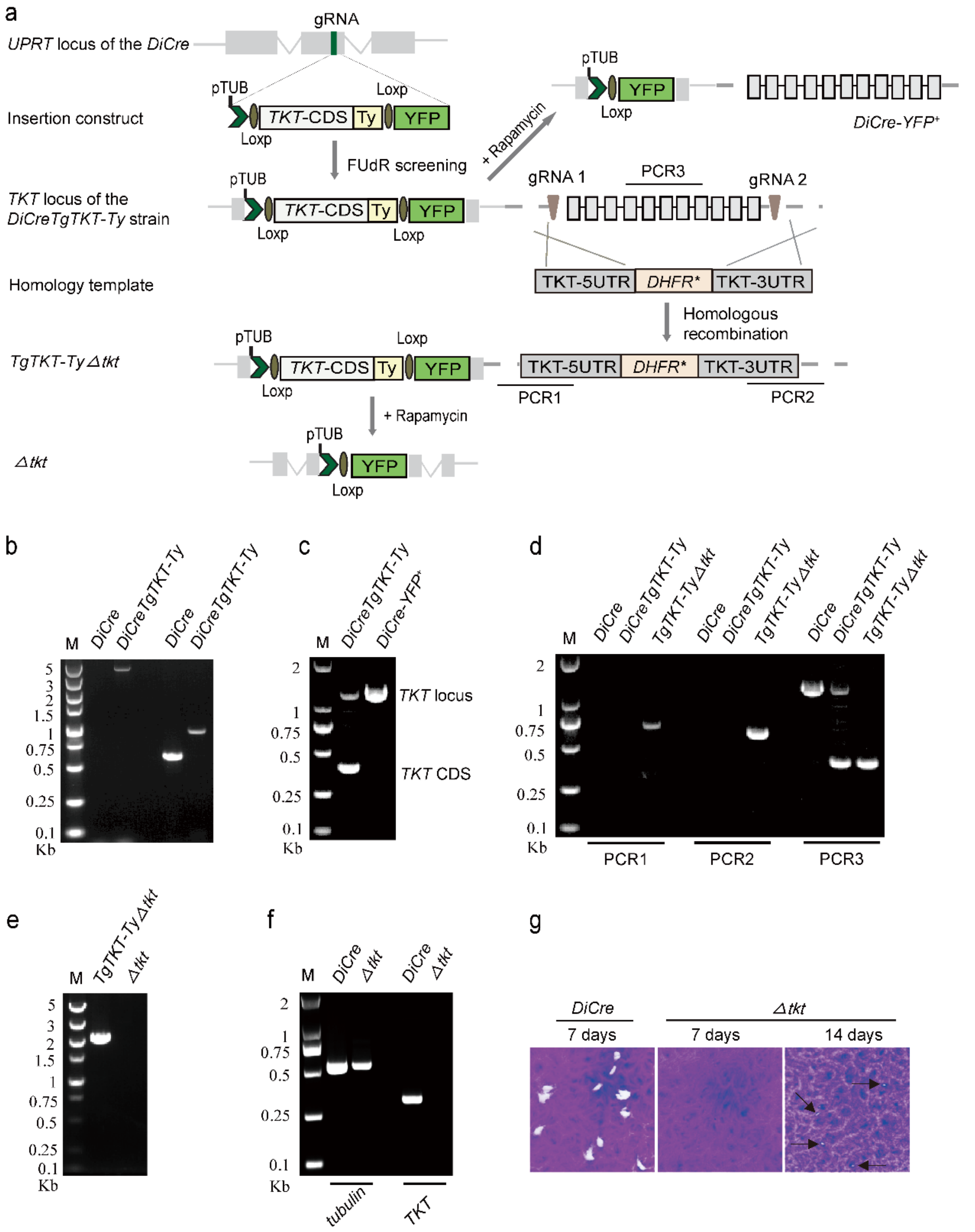

Supplementary Fig. 6: Construction and phenotypic characterization of the TKT mutants.

a Schematics of TKT mutants. Firstly, C-terminally Ty-tagged ORF of *TKT* was integrated at the *UPRT* locus by 5-fluorodeoxyuridine (FUdR) selection, and then the *TKT* locus was replaced by *DHFR-TS** selection marker using CRISPR/Cas9-assisted gene replacement in the *DiCreTgTKT-Ty* strain. Subsequently, *DiCreTgTKT-Ty* and *TgTKT-TyΔtkt* strains were treated with rapamycin for 4 days and then YFP reporter strain (*DiCre-YFP*$^{+}$) and *TKT*-knockout (*Δtkt*) clonal mutants were isolated by limited dilution in 96-well plates. **b-e** Diagnostic PCRs confirming the *DiCreTgTKT-Ty*,

DiCre-YFP^{+}, *TgTKT-TyΔtkt* and *Δtkt* strains. **f** Semi-quantitative RT-PCR of the *Δtkt* mutant (tubulin, control). **g** Plaques formed by the *Δtkt* and *DiCre* (parental) strains cultured in standard medium for 7 or 14 days. Black arrows indicate minuscule plaques of the *Δtkt* mutant on day 14. Source data are provided as a Source data file.

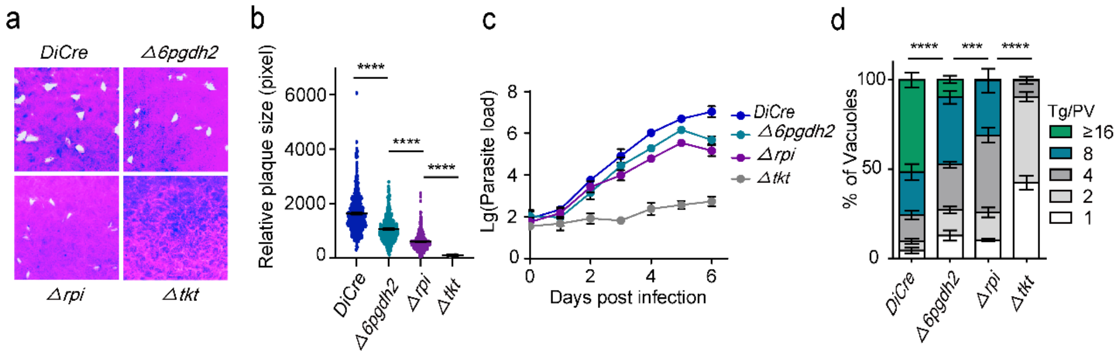

Supplementary Fig. 7: Comparative growth of the *Δ6pgdh2*, *Δrpi* and *Δtkt* mutants.

a-b Plaque assay (n = 3 experiments, means ± SEM; ****, $p < 0.0001$, unpaired two-tailed Student's *t*-test). **c** Growth curve analysis. n = 2 experiments, means ± SEM. **d** Intracellular replication assay (24 h). The number of parasites in PV was quantified after immunostaining (n = 3 assays, means ± SEM; ***, $p = 0.0006$, ****, $p < 0.0001$, two-way ANOVA). Source data are provided as a Source data file.

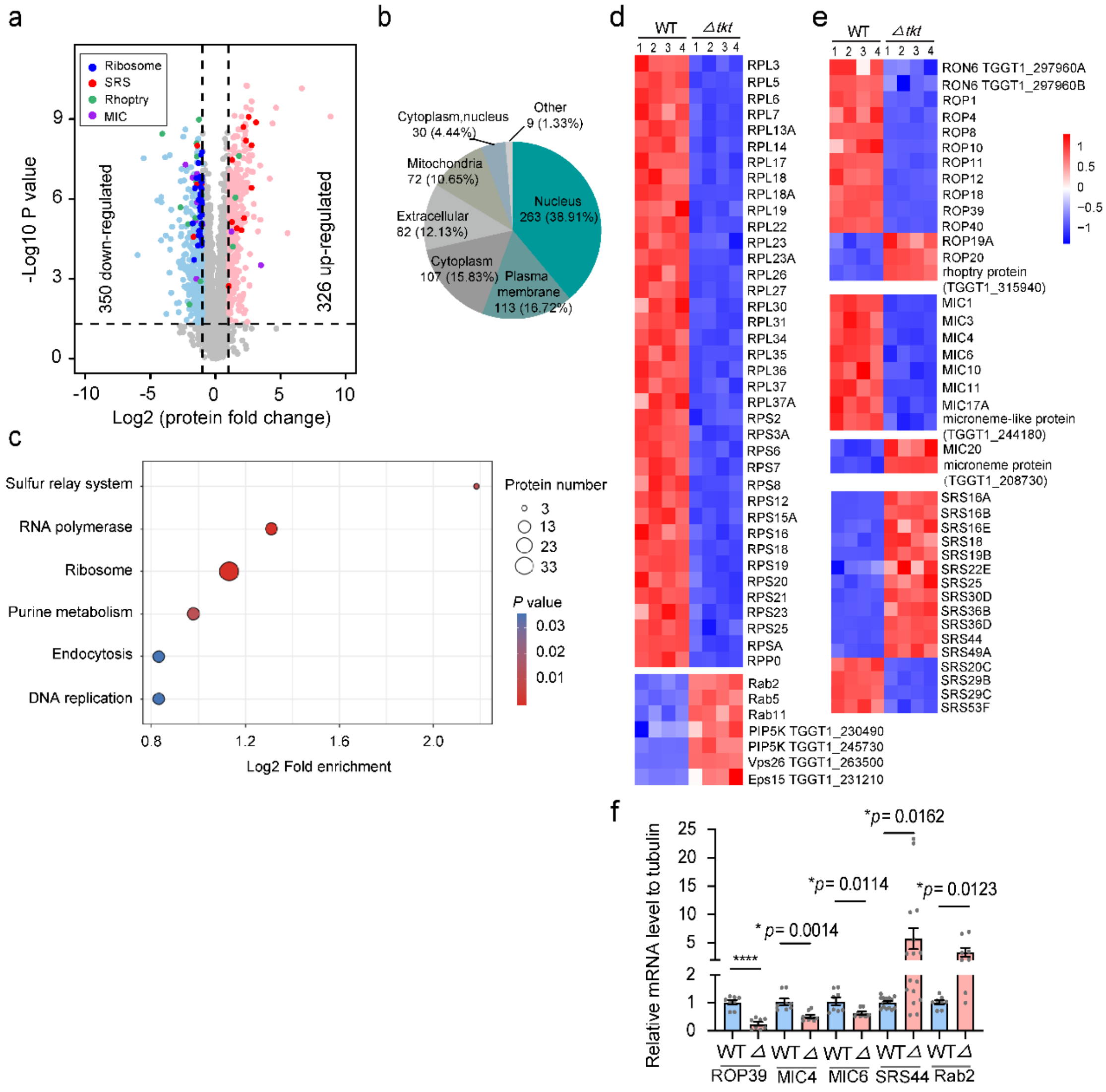

Supplementary Fig. 8: *TKT* deletion alters the proteome of the mutant.
a Proteomic profile of the *Δtkt* strain. Volcano plot illustrates ribosome, rhoptry, micronemal and stage-specific SRS Proteins in blue, green, purple and red color, respectively. **b** Pie chart revealing the subcellular localization of differentially expressed proteins. **c** KEGG analysis of proteins with Log2 fold changes ≥ 2 and $p \leq 0.05$. **d-e** Heatmap based on differential expression of ribosomal proteins, Rab and PIP5K proteins, microneme, rhoptry, and stage-specific proteins. **f** Quantitative RT-PCR validation of indicated transcripts. β-tubulin was used as an internal reference (means ± SEM; ****, $p < 0.0001$, unpaired two-tailed Student's t-test). Source data are provided as a Source data file.

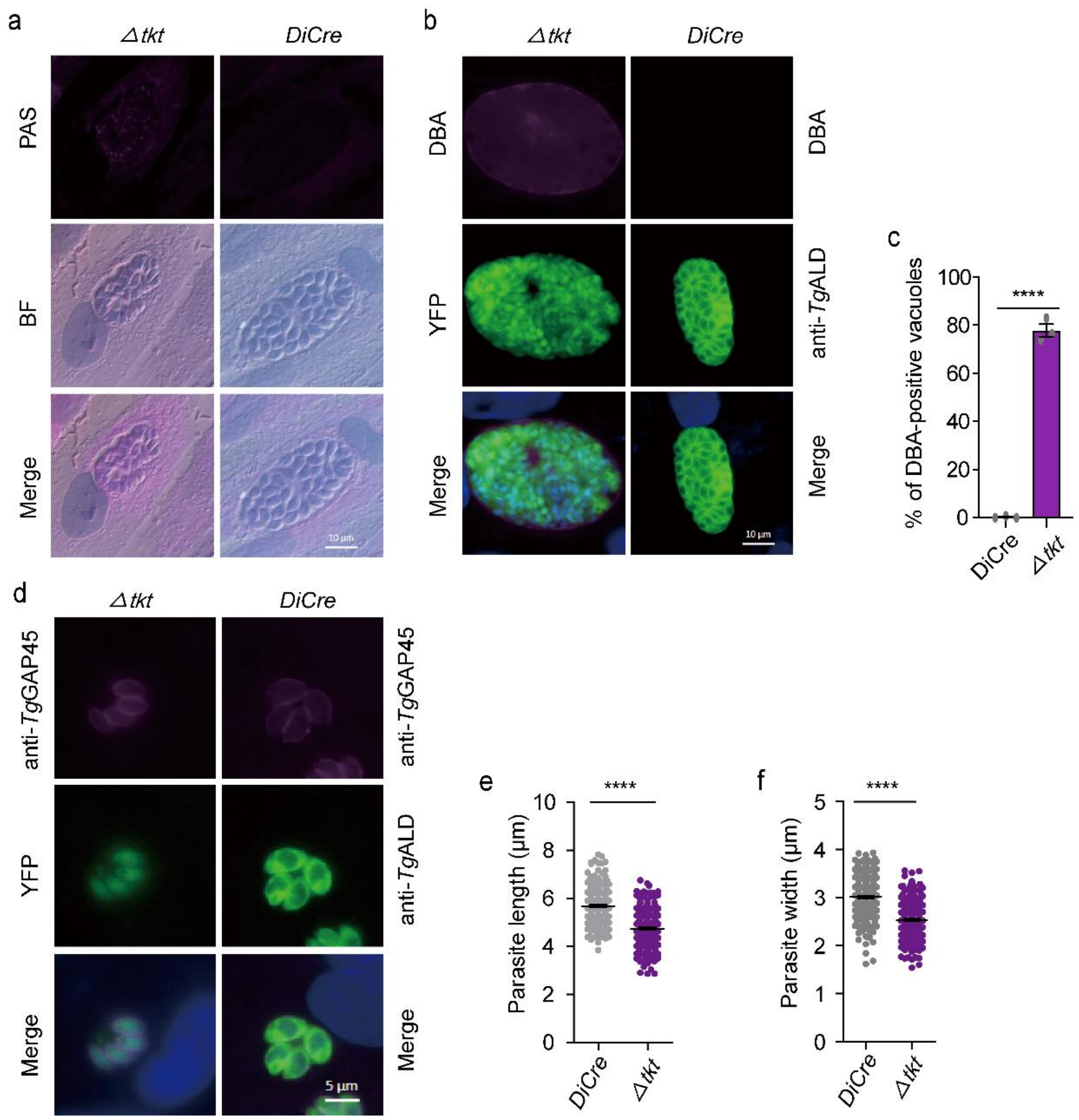

Supplementary Fig. 9: TKT is essential for starch metabolism and bradyzoite differentiation.

a Periodic Acid-Schiff (PAS) staining of the *DiCre* and *Δtkt* strains to visualize amylopectin granules (magenta). Scale bars = 10 µm. **b** Cyst wall staining by Dolichos Biflorus Agglutinin (DBA, magenta). Anti-*Tg*ALD antibody (green) and YFP (green) were used to detect parasites**.** Scale bars = 10 µm. **c** DBA-positive vacuoles containing 4 or more parasites. A minimum of 100 vacuoles were examined (n = 3 assays. means ± SEM; ****, $p < 0.0001$, unpaired two-tailed Student's *t*-test). **d** The parental (*DiCre*) strain was stained with the mouse anti-*Tg*GAP45 and rabbit anti-*Tg*ALD antibodies, whereas the *Δtkt* strain (YFP-positive) was labeled with the mouse anti-*Tg*GAP45 antibody. Scale bars = 5 µm. **e-f** Morphometric dimensions of the *Δtkt* and *DiCre* strains. Parasitophorous vacuoles harboring 4 parasites were analyzed for the length and width of parasites (means ± SEM of > 200 parasites, ****, $p < 0.0001$, unpaired two-tailed Student's *t*-test). Source data are provided as a Source data file.

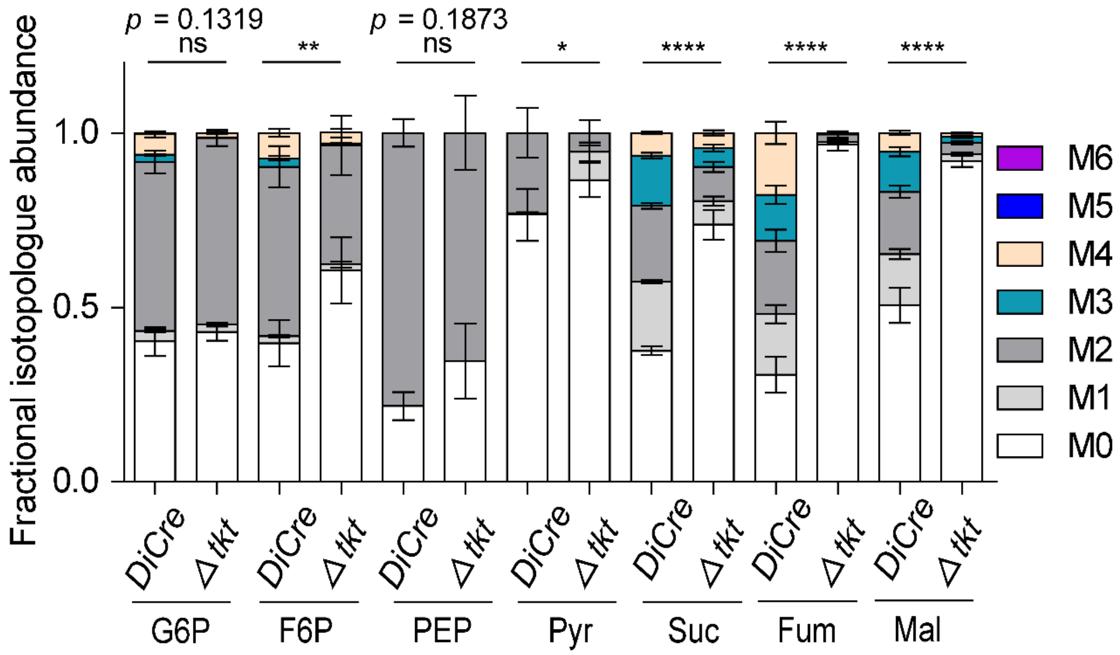

Supplementary Fig. 10: Metabolic alterations in the TKT-null mutant.

Extracellular parasites of the *DiCre* (parental) and *Δtkt* strains were incubated for 4 h in a medium with 8 mM 1,2-$^{13}C_2$-glucose. The incorporation of ^{13}C into glycolysis and TCA cycle intermediates was measured by UHPLC-HRMS. G6P: glucose-6-phosphate; F6P: fructose-6-phosphate; PEP: phosphoenolpyruvate; Pyr: pyruvate; Suc: succinate; Fum: fumarate; Mal: malate. M0-M6 represents the number of carbon atoms in selected ^{13}C-labeled metabolites (n = 5 experiments, means ± SEM; ns, not significant, *, $p = 0.0144$, **, $p = 0.0099$, ****, $p < 0.0001$, two-way ANOVA). Source data are provided as a Source data file.
