# Supplementary material for: Metabolic plasticity, essentiality and therapeutic potential of ribose-5-phosphate synthesis in Toxoplasma gondii
Source: Nat Commun. 2024 Apr 8;15:2999. doi: 10.1038/s41467-024-47097-8 (PMC11001932; doi:10.1038/s41467-024-47097-8)
Supplement: Supplementary file 3 — Description of Additional Supplementary Files [file 41467_2024_47097_MOESM3_ESM.docx]

**Description of Additional Supplementary Files**

**File Name: Supplementary Data 1**
Description: Oligonucleotides used in this study.

**File Name: Supplementary Data 2**
Description: Plasmids used in this study.

**File Name: Supplementary Data 3**
Description: Parental and derivative strains used in this study.

**File Name: Supplementary Data 4**
Description: The transcriptome data of *DiCre* and *ΔsbpaseΔtal* strains

**File Name: Supplementary Data 5**
Description: The metabolomics dataset of intracellular parasites of the *DiCre* and *ΔsbpaseΔtal* strains incubated for 12 h in a medium with 8 mM 1,2-^13^C_2_-glucose.

**File Name: Supplementary Data 6**
Description: The metabolomics dataset of extracellular parasites of the *DiCre* and *Δtkt* strains incubated for 4 h in a medium with 8 mM 1,2-^13^C_2_-glucose.

**File Name: Supplementary Data 7**
Description: The metabolomics dataset of intracellular parasites of the *DiCre* and *Δtkt* strains grown under standard tissue culture conditions.

**File Name: Supplementary Data 8**
Description: The metabolomics dataset of intracellular parasites of the *DiCre* and *Δtkt* strains incubated for 12 h in a medium with 8 mM 1,2-^13^C_2_-glucose.

**File Name: Supplementary Data 9**
Description: Relative levels of AMP and IMP in *DiCre* and *ΔsbpaseΔtal* parasites.

**File Name: Supplementary Data 10**
Description: The proteomics dataset of WT and *Δtkt* strains
