# Supplementary material for: Metabolic plasticity, essentiality and therapeutic potential of ribose-5-phosphate synthesis in Toxoplasma gondii
Source: Nat Commun. 2024 Apr 8;15:2999. doi: 10.1038/s41467-024-47097-8 (PMC11001932; doi:10.1038/s41467-024-47097-8)
Supplement: Supplementary file 14 — Reporting Summary [file 41467_2024_47097_MOESM14_ESM.pdf]

nature portfolio

Corresponding author(s): Ningbo Xia

Last updated by author(s): Feb 26, 2024

# Reporting Summary

Nature Portfolio wishes to improve the reproducibility of the work that we publish. This form provides structure for consistency and transparency in reporting. For further information on Nature Portfolio policies, see our Editorial Policies and the Editorial Policy Checklist.

## Statistics

For all statistical analyses, confirm that the following items are present in the figure legend, table legend, main text, or Methods section.

| n/a | Confirmed | |
|---|---|---|
| ☐ | ☒ | The exact sample size (*n*) for each experimental group/condition, given as a discrete number and unit of measurement |
| ☐ | ☒ | A statement on whether measurements were taken from distinct samples or whether the same sample was measured repeatedly |
| ☐ | ☒ | The statistical test(s) used AND whether they are one- or two-sided<br>*Only common tests should be described solely by name; describe more complex techniques in the Methods section.* |
| ☒ | ☐ | A description of all covariates tested |
| ☐ | ☒ | A description of any assumptions or corrections, such as tests of normality and adjustment for multiple comparisons |
| ☐ | ☒ | A full description of the statistical parameters including central tendency (e.g. means) or other basic estimates (e.g. regression coefficient) AND variation (e.g. standard deviation) or associated estimates of uncertainty (e.g. confidence intervals) |
| ☐ | ☒ | For null hypothesis testing, the test statistic (e.g. *F*, *t*, *r*) with confidence intervals, effect sizes, degrees of freedom and *P* value noted<br>*Give P values as exact values whenever suitable.* |
| ☒ | ☐ | For Bayesian analysis, information on the choice of priors and Markov chain Monte Carlo settings |
| ☒ | ☐ | For hierarchical and complex designs, identification of the appropriate level for tests and full reporting of outcomes |
| ☒ | ☐ | Estimates of effect sizes (e.g. Cohen's *d*, Pearson's *r*), indicating how they were calculated |

*Our web collection on statistics for biologists contains articles on many of the points above.*

## Software and code

Policy information about availability of computer code

| | |
|---|---|
| Data collection | ZEN 2 was used to collect IFA images; HTC1 1.5.0.6 for qPCR test. ScanWizardEZ for plaques capture. Gen5 CHS 3.03 for ATP assays. VisionWorks for PCR images. NIS-Elements D 5.30.00 64 for YFP-fluorescence observed. RNA-seq sequencing library was sequenced with the Illumina NovaSeq 6000 sequencer; The UPLC-MS data were pocessed using Q Exactive Hybrid Quadrupole-Orbitrap Mass Spectrometryusing; Xcalibur software (version 3.0.63) ;IsoCor v2; Whole genome sequencing was on Illumina NovaSeq6000; Integrative Genomics Viewer 2.16.2; The CytExpert 2.0.0.153 was used for flow cytometry analyses; BD FACSMelody for flow separation. Proteomics data was acquired by timsTOF Pro mass spectrometry. |
| Data analysis | Microsoft Excel for Mac; GraphPad Prism 8.0.1; SnapGene 4.3.6; Adobe Illustrator 2021; Adobe Photoshop CC 2019; HTC1 1.5.0.6; IVIS Spectrum imaging system; cellSens software; IVIS Spectrum imaging system; multi-mode plate reader;HISTA2;RSEM;Deseq2;Integrative Genomics Viewer 2.16.2. |

For manuscripts utilizing custom algorithms or software that are central to the research but not yet described in published literature, software must be made available to editors and reviewers. We strongly encourage code deposition in a community repository (e.g. GitHub). See the Nature Portfolio guidelines for submitting code & software for further information.

## Data

Policy information about availability of data

All manuscripts must include a data availability statement. This statement should provide the following information, where applicable:

- Accession codes, unique identifiers, or web links for publicly available datasets
- A description of any restrictions on data availability
- For clinical datasets or third party data, please ensure that the statement adheres to our policy

The genome sequencing data have been deposited and released with the accession number PRJNA947326 (https://www.ncbi.nlm.nih.gov/bioproject/PRJNA947326/). Transcriptomics sequencing data are available in the short read archive (SRA) of the database of the National Center for Biotechnology Information under the accession number PRJNA947324(https://www.ncbi.nlm.nih.gov/bioproject/PRJNA947324/). The raw proteomics data have been deposited in the ProteomeXchange Consortium (www.proteomexchange.org) via the iProX partner repository with the accession number: PDX041014. All other data are presented in the article or the supplementary information. Source data are provided with this paper. Reference genome of the Toxoplasma GT1 strain: https://toxodb.org/toxo/app/record/dataset/NCBITAXON_507601.

## Research involving human participants, their data, or biological material

Policy information about studies with human participants or human data. See also policy information about sex, gender (identity/presentation), and sexual orientation and race, ethnicity and racism.

| | |
|---|---|
| Reporting on sex and gender | N/A |
| Reporting on race, ethnicity, or other socially relevant groupings | N/A |
| Population characteristics | N/A |
| Recruitment | N/A |
| Ethics oversight | N/A |

Note that full information on the approval of the study protocol must also be provided in the manuscript.

# Field-specific reporting

Please select the one below that is the best fit for your research. If you are not sure, read the appropriate sections before making your selection.

☒ Life sciences ☐ Behavioural & social sciences ☐ Ecological, evolutionary & environmental sciences

For a reference copy of the document with all sections, see nature.com/documents/nr-reporting-summary-flat.pdf

# Life sciences study design

All studies must disclose on these points even when the disclosure is negative.

| | |
|---|---|
| Sample size | Since the study involves comparison between wild type parasites and knockout mutant parasites, a minimum of three biological replicates were chosen for each separate experiment. Sample sizes in other experiments were chosen to reflect biological and technical variance of the investigated parameters based based on previously published literature (1).<br>1. Jin-Lei Wang, Ting-Ting Li, Hany M. Elsheikha, Qin-Li Liang,Zhi-Wei Zhang, MengWang, L. David Sibley, Xing-Quan Zhu. The protein phosphatase 2A holoenzyme is a key regulator of starch metabolism and bradyzoite differentiation in Toxoplasma gondii. Nature Communications. 2022 Dec 8;13(1):7560. |
| Data exclusions | For virulence test, blood samples collected from surviving animals were tested 30 days post-infection, and mice seronegative by immunofluorescence were excluded from further analysis. |
| Replication | All experiments were repeated at least with 2-3 biological replicates. All results were successfully replicated.<br>In vivo experiment, mouse numbers (n = 3 - 10 in each group) were based on our previously published work and elsewhere in the literature. |
| Randomization | Mice were randomly assigned to experimental groups. Randomization does not apply to our other experiments as we have been analyzing wild type parasites vs knock-out mutants, or experiments conducted by treating samples and controls side by side under the indicated conditions |
| Blinding | The plaque assays were performed in a blinded manner and confirmed independently by two different people. For other experiments, blinding was not needed because the data produced had clear endpoints that were not subjected to investigator bias. |

# Reporting for specific materials, systems and methods

We require information from authors about some types of materials, experimental systems and methods used in many studies. Here, indicate whether each material, system or method listed is relevant to your study. If you are not sure if a list item applies to your research, read the appropriate section before selecting a response.

## Materials & experimental systems

| n/a | Involved in the study |
|---|---|
| ☐ | ☒ Antibodies |
| ☐ | ☒ Eukaryotic cell lines |
| ☒ | ☐ Palaeontology and archaeology |
| ☐ | ☒ Animals and other organisms |
| ☒ | ☐ Clinical data |
| ☒ | ☐ Dual use research of concern |
| ☒ | ☐ Plants |

## Methods

| n/a | Involved in the study |
|---|---|
| ☒ | ☐ ChIP-seq |
| ☐ | ☒ Flow cytometry |
| ☒ | ☐ MRI-based neuroimaging |

## Antibodies

Antibodies used

Anti-HA-tag mAb, MBL, Cat#M180-3, RRID: AB_10951811 (diluted 1:1000)
Dolichos Biflorus Agglutinin (DBA), Rhodamine, Vectorlabs, Cat# RL-1032-2, (diluted 1:100)
Dolichos Biflorus Agglutinin (DBA), Fluorescenin, Vectorlabs, Cat# FL-1031-2, (diluted 1:100)
Anti-mouse IgG (H+L), F(ab)2 Fragment (Alexa Fluor 594 Conjugate), Cell Signaling Technology, Cat#8890, RRID: AB_2714182, (diluted 1:1000)
Anti-rabbit IgG (H+L), F(ab)2 Fragment (Alexa Fluor 594 Conjugate), Cell Signaling Technology, Cat#8889, RRID: AB_2716249, (diluted 1:1000)
Anti-mouse IgG (H+L), F(ab)2 Fragment (Alexa Fluor 488 Conjugate), Cell Signaling Technology, Cat#4408, RRID: AB_1904020, (diluted 1:1000)
Anti-rabbit IgG (H+L), F(ab)2 Fragment (Alexa Fluor 488 Conjugate), Cell Signaling Technology, Cat#4412, (diluted 1:1000)

Validation

Anti-HA-tag mAb (1:1000) (MBL; Cat# M180-3): Validated for IFA: Cui CP et al. Dynamic ubiquitylation of Sox2 regulates proteostasis and governs neural progenitor cell differentiation. Nat Commun. 9 4648 (2018). Du T et al. The deubiquitylase OTUD3 stabilizes GRP78 and promotes lung tumorigenesis. 10, 2914 (2019).
Rabbit anti-ALD (1:1000): Xia NB, Guo XF, Guo QH, Gupta N, Ji N, Shen B, Xiao LH, Feng YY. Metabolic flexibilities and vulnerabilities in the pentose phosphate pathway of the zoonotic pathogen Toxoplasma gondii. PLoS Pathog. 2022; 18, e1010864.
Mouse anti-GAP45 (1:1000): Validated by immunofluorescence staining of the parasites: Wang Jin-Lei, Li Ting-Ting, Zhang Nian-Zhang et al. The transcription factor AP2XI-2 is a key negative regulator of Toxoplasma gondii merogony. Nat Commun, 2024, 15: 793.
Rabbit anti-HSP60 (1:100): Validated by immunofluorescence staining of the tachyzoites.
Mouse anti-SBPase (1:1000): Produced in this study. Validated by immunofluorescence staining of the tachyzoites.
Mouse anti-TAL (1:1000): Produced in this study. Validated by immunofluorescence staining of the tachyzoites.
Mouse anti-TKT (1:1000): Produced in this study. Validated by immunofluorescence staining of the tachyzoites.
Mouse anti-Ty (1:1000): Validated by immunofluorescence staining of the tachyzoites.(mBio. 2017 May 2;8(3):e00375-17.)
Rabbit anti-ALD (1:1000), Mouse anti-GAP45 (1:1000), Rabbit anti-HSP60 (1:100), Mouse anti-Ty (1:1000) were provided by Bang Shen (Huazhong Agricultural University in Wuhan).

## Eukaryotic cell lines

Policy information about cell lines and Sex and Gender in Research

Cell line source(s)

HFF (primary human foreskin fibroblast) was obtained from ATCC (SCRC-1041).

Authentication

Cells lines were authenticated by visual inspection of morphology.

Mycoplasma contamination

Cell culture samples were tested and confirmed negative for Mycoplasma.

Commonly misidentified lines (See ICLAC register)

No commonly misidentified cell lines were used in this study

## Animals and other research organisms

Policy information about studies involving animals; ARRIVE guidelines recommended for reporting animal research, and Sex and Gender in Research

Laboratory animals

Seven-week-old female ICR mice were purchased from the Guangdong Medical Experimental Animal Center (Guangdong Province), while Balb/c-nu mice were acquired from Guangzhou Ruige Biotechnology Co., LTD. Mice were hosted in clean filter-top cages with a 12:12 light-dark cycle, 50–60% humidity, and 22°C room temperature and raised following standard protocols as per the regulations

| | |
|---|---|
| | of the Administration of Affairs Concerning Experimental Animals (Permit 2022f210, the Ethical Committee of South China Agricultural University,Guangzhou). |
| Wild animals | No wild animals were used in the study. |
| Reporting on sex | Mice using in the study are all female. |
| Field-collected samples | No field collected samples were used in the study. |
| Ethics oversight | Animal studies are approved by the Administration of Affairs Concerning Experimental Animals (Permit 2022f210, the Ethical Committee of South China Agricultural University, Guangzhou). |

Note that full information on the approval of the study protocol must also be provided in the manuscript.

# Flow Cytometry

## Plots

Confirm that:

- [x] The axis labels state the marker and fluorochrome used (e.g. CD4-FITC).
- [x] The axis scales are clearly visible. Include numbers along axes only for bottom left plot of group (a 'group' is an analysis of identical markers).
- [ ] All plots are contour plots with outliers or pseudocolor plots.
- [x] A numerical value for number of cells or percentage (with statistics) is provided.

## Methodology

| | |
|---|---|
| Sample preparation | *Describe the sample preparation, detailing the biological source of the cells and any tissue processing steps used.* |
| Instrument | *Identify the instrument used for data collection, specifying make and model number.* |
| Software | *Describe the software used to collect and analyze the flow cytometry data. For custom code that has been deposited into a community repository, provide accession details.* |
| Cell population abundance | *Describe the abundance of the relevant cell populations within post-sort fractions, providing details on the purity of the samples and how it was determined.* |
| Gating strategy | *Describe the gating strategy used for all relevant experiments, specifying the preliminary FSC/SSC gates of the starting cell population, indicating where boundaries between "positive" and "negative" staining cell populations are defined.* |

- [x] Tick this box to confirm that a figure exemplifying the gating strategy is provided in the Supplementary Information.
